# Unexpected right-handed helical nanostructures co-assembled from l-phenylalanine derivatives and achiral bipyridines†
†Electronic supplementary information (ESI) available: Additional characterization figures. See DOI: 10.1039/c6sc04808k


**DOI:** 10.1039/c6sc04808k

**Published:** 2017-01-04

**Authors:** Guofeng Liu, Jinying Liu, Chuanliang Feng, Yanli Zhao

**Affiliations:** a State Key Lab of Metal Matrix Composites , School of Materials Science and Engineering , Shanghai Jiao Tong University , 800 Dongchuan Road , Shanghai , 200240 , China . Email: clfeng@sjtu.edu.cn; b Division of Chemistry and Biological Chemistry , School of Physical and Mathematical Sciences , Nanyang Technological University , 21 Nanyang Link , 637371 , Singapore . Email: zhaoyanli@ntu.edu.sg; c School of Materials Science and Engineering , Nanyang Technological University , 50 Nanyang Avenue , 639798 , Singapore

## Abstract

The construction of chiral supramolecular systems with desirable handedness is of great importance in materials science, chemistry, and biology since chiral nanostructures exhibit fascinating photophysical properties and unique biological effects. Herein, we report that achiral bipyridines can co-assemble with l-phenylalanine derivatives into unexpected right-handed helical nanostructures rather than a left-handed helix *via* intermolecular hydrogen bonding interactions formed between the pyridyl and carboxylic groups. This study opens up a route to develop chiral nanostructures with desirable handedness *via* the co-assembly of simple molecular building blocks and provides a straightforward insight into the chirality control of nanostructures in supramolecular systems.

## Abstract 2

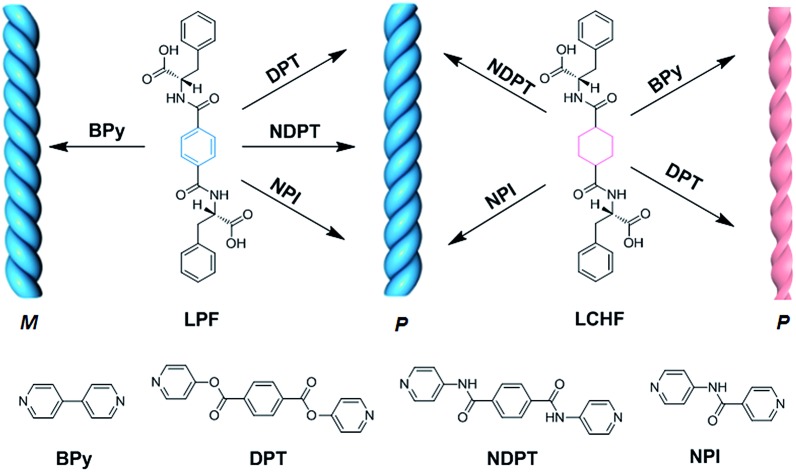
Achiral bipyridines can co-assemble with l-phenylalanine derivatives into unexpected right-handed helical nanostructures rather than left-handed helix by utilizing intermolecular hydrogen bonding formed between pyridyl and carboxylic groups.

## Introduction

Chiral supramolecular architectures assembled from small molecular building blocks have been attracting extensive interest due to their controllable structural features, their relationship to biological structures, and potential applications in chiral recognition and separation.^1^ Although numerous helical or twisted nanostructures and ordered ensembles have been successfully produced by molecular self-assembly from either single or multiple molecular components for utilization in chemistry,^2^ biology,^3^ and materials science,^4^ it still remains a challenge to construct chiral nanostructures with desirable conformation (*i.e.*, right-handed, P; left-handed, M) from specific chiral building blocks at will. On the other hand, in comparison with the rich knowledge that has been gathered with regard to establishing chiral nanostructures from either chiral or achiral building blocks,^5^ rare studies have reflected an explicit relationship between the chirality of nanoarchitectures and enantiomeric monomers.^6^ Obviously, gel-phase materials are a key test-bed for understanding the impact of molecular chirality on nanoscale self-assembly or co-assembly since supramolecular chirality of gels can be finely tuned by both the chirality of component molecules^7^ and special spatial arrangements of building blocks.^8^ After a detailed survey of previous studies, it was found that left-handed twist or helix is often co- or self-assembled from l-form amino acid-based molecular building blocks, whereas right-handed twist or helix is normally aggregated from d-type counterparts.^9^ There are some reports on right-handed twist or helix co-assembled from l-type amino acid-based building blocks.8c,^10^ Thus, how to fine tailor the building blocks aggregation into a desirable specific motif is early stage study.

**Fig. 1:**
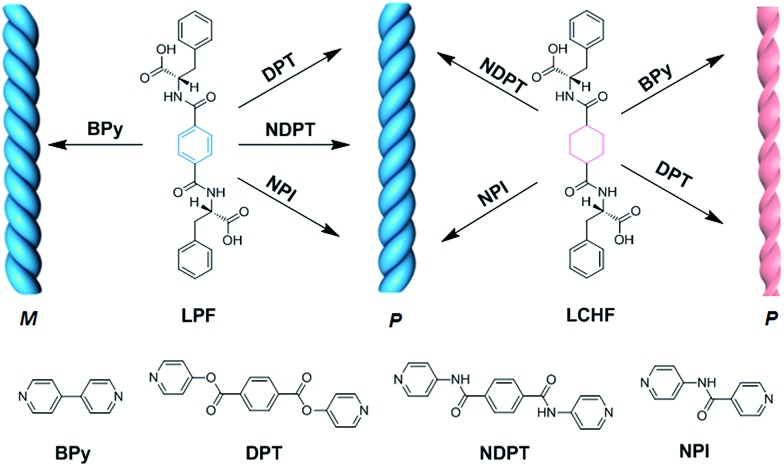
Schematic of chiral nanostructures co-assembled from l-type enantiomeric monomers (LPF and LCHF) with achiral bipyridines (BPy, DPT, NDPT, and NPI). M and P denote left- and right-handed helical nanostructures, respectively.

It is important to gain further insights into the fundamentals of chiral transfer and expression in the co-assembled hydrogel systems, which will enable us to obtain a comprehensive understanding of the design of new chiral materials and to fine tune the chirality of the co-assemblies. Herein, uniform right-handed helical nanostructures were obtained from the co-assembly of various achiral bipyridine derivatives with two chiral gelators (LPF and LCHF) derived from l-phenylalanine through strong intermolecular hydrogen bonding formed between achiral bipyridines and l-type enantiomers (Fig. 1).

## Results and discussion

### Unexpected right-handed helical nanostructures of hydrogels

Molecules LPF and LCHF, which are based on 1,4-phenyldicarboxamide and 1,4-cyclohexanedicarboxamide respectively, contain a helicogenic l-phenylalanine motif and carry a COOH group at each terminus of the two phenylalanine arms. Hence, they are bistopic ligands. To co-assemble them into hydrogels, we rationally designed four bistopic bipyridine ligands (BPy, DPT, NDPT, and NPI) by utilizing hydrogen-bonding interactions between the pyridyl nitrogen atom and the H–O group of carboxylic acid.^11^ The synthesis of LCHF, DPT, and NPI has been outlined in the Experimental section, and BPy was commercially available. LPF and NDPT were synthesized according to a previous report.8*c* All the newly synthesized compounds were fully characterized by NMR spectroscopy and high-resolution mass spectrometry (Fig. S1–S14†).

**Fig. 2:**
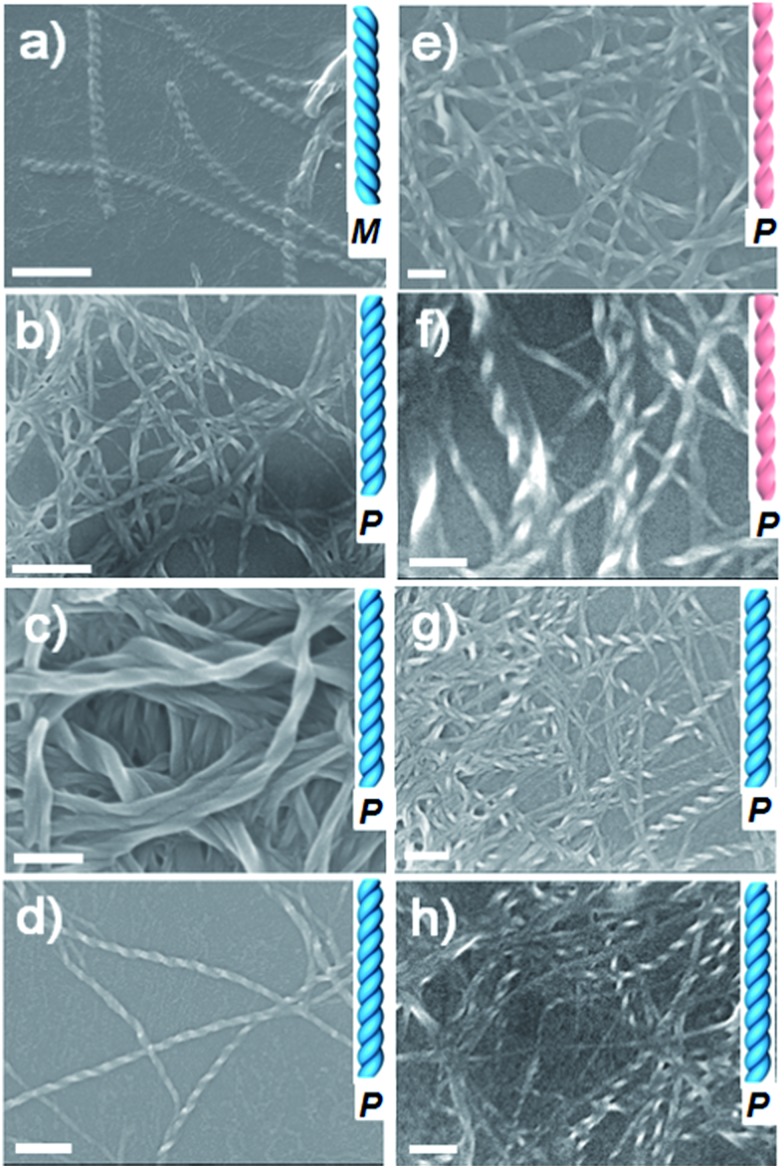
SEM images of supramolecular hydrogels based on LPF or LCHF co-assembled with achiral bipyridines. (a) LPF + BPy, (b) LPF + DPT, (c) LPF + NDPT, (d) LPF + NPI, (e) LCHF + BPy, (f) LCHF + DPT, (g) LCHF + NDPT, and (h) LCHF + NPI. Scale bars: 5 μm for (a), 500 nm for (b), and 200 nm for (c–h).

The ability of four achiral bipyridines (BPy, DPT, NDPT, and NPI) to co-assemble with equimolar LPF or LCHF was first determined by the formation of hydrogels by means of heating-to-cooling and inversion tests (Fig. S15†). LPF + DPT, LPF + NPI, LPF + BPy, and LPF + NDPT formed stable homogeneous hydrogels in the vials. Scanning electron microscopy (SEM) images of the diluted samples of gels on a silicon wafer showed enantiomerically enriched, helical ribbon fibers (Fig. 2 and S16–S23†). All the co-assembled hydrogels (Fig. 2a–d) were organized into rope-like fibers with the helicity pitches around hundreds of nanometers. In Fig. 2a, the fibers from the LPF + BPy gel exhibited exclusively left-handed (M-type) helicity with diameter in hundreds of nanometers. Surprisingly, fibers from LPF + DPT, LPF + NPI, and LPF + NDPT (Fig. 2b–d) all displayed a beautiful uniform right-handed (P-type) helix with diameter in tens of nanometers, which had absolutely opposite chirality of the LPF + BPy gel. According to previous reports,3a,9a–c specific one-dimensional nanofibers self-assembled from l-phenylalanine derived monomers usually exhibit exact left-handedness.

To explore if this unexpected phenomenon is also applicable to other gel systems, microscopic nanostructures assembled from achiral bipyridines with LCHF were investigated in detail. Intriguingly, uncommon right-handed helical nanofibers with nearly the same helicity pitches around hundreds of nanometers were observed in the SEM images of all hydrogels (Fig. 2e–h). This is a bit different with LPF-based gel systems, where LPF + BPy exhibited exclusively left-handed (M-type) helicity. However, right-handed nanofibers were observed from LCHF + BPy. On the basis that the only difference in LCHF + BPy is the central benzene ring of LPF instead of the cyclohexyl core of LCHF, it was anticipated that the chirality of supramolecular aggregates could be tuned by only changing some functional groups rather than altering the inherent chirality of enantiomeric monomers. In addition, these studies indicated how a slight change in the molecular structure of the building blocks dramatically influences the overall chirality of the supramolecular aggregates in two-component hydrogels. The chirality of the assemblies shown herein is not only strongly determined by the chiral center of the phenylalanine units in LPF and LCHF, but is also highly affected by the molecular structure of achiral bipyridines, both of which collectively play vital roles in rigidifying the aggregates and guiding them to form unique chiral hydrogels.

### CD activity of co-assembled hydrogels

**Fig. 3:**
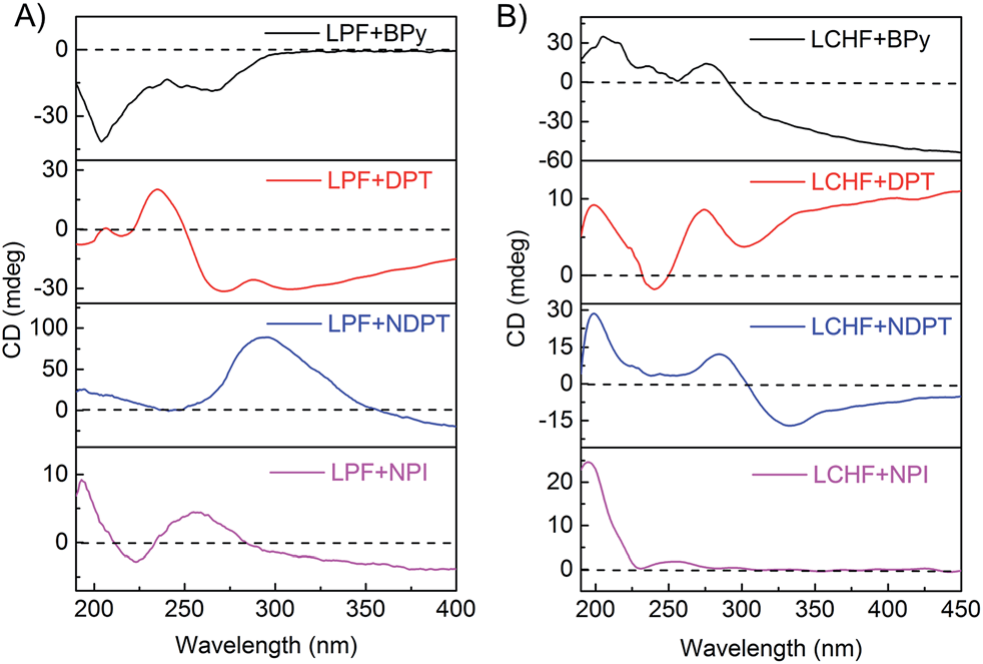
CD spectra of hydrogels for (A) LPF + BPy, LPF + DPT, LPF + NDPT, and LPF + NPI, and (B) LCHF + BPy, LCHF + DPT, LCHF + NDPT, and LCHF + NPI.

To gain further insight into these helical supramolecular structures, circular dichroism (CD) spectra of the co-assembled hydrogels were obtained at room temperature (Fig. 3 and S24†). The CD spectra of hydrogels LPF + NPI, LPF + DPT, and LPF + BPy all exhibited a negative dichroic signal at around 205 nm that was assigned to intramolecular π–π* transitions in the peripheral phenyl group of LPF.8*c* Interestingly, for LPF + BPy hydrogels the CD spectrum also showed a negative Cotton effect at 268 nm (Fig. 3A), whereas for LPF + DPT, LPF + NDPT, and LPF + NPI hydrogels, a positive dichroic signal was observed at 235 nm, 293 nm, and 265 nm, respectively, which was assigned to the intramolecular transitions from the amide linkage to the central aryl group according to our previous calculations on LPF.8*c* Compared with the hydrogels of LPF + BPy, a chiral transition into opposite optically active hydrogels (LPF + DPT, LPF + NDPT, and LPF + NPI) was obtained only by changing the achiral components (BPy, DPT, NDPT, and NPI), which correlated well with the helical microscopic structures observed in the SEM images. In addition, the contribution of linear dichroism (LD) on the CD signals was investigated. As shown in Fig. S25,† the intensity of LD signals was much lower than that of the corresponding CD signals (except for LPF + NPI), indicating that the LD contribution could be negligible in these gels. In the case of LPF + NPI hydrogel, to get rid of the LD influence, the hydrogel film was placed at different angles, and an average CD signal (Fig. S26†) was obtained using a method reported in the literature.^12^

Thus, the relationship between the handedness of the helical fibers and CD signals could be obtained. The M-type LPF + BPy co-assembly exhibited a negative Cotton effect at 268 nm, whereas P-type supramolecular structures in LPF + DPT, LPF + NDPT, and LPF + NPI showed a positive dichroic signal (Fig. 3A). Note that all the hydrogels possessed the same S-type stereocenter within the peripheral l-phenylalanine units. Thus, their CD spectra should not be the exact mirror images. However, the chirality of these supramolecular assemblies and their chiroptical activities could be inversed by altering the achiral bipyridines. For the LCHF systems, all the CD spectra (Fig. 3B) of hydrogels (LCHF + BPy, LCHF + DPT, LCHF + NDPT, and LCHF + NPI) exhibited positive dichroic signals around 200 nm and among 250–283 nm from the amide linkage, which are in good agreement with the right-handed helical nanofibers observed in the SEM images (Fig. 2e–h). The enantiomer of LCHF, *i.e.*, DCHF was also synthesized as a control. The corresponding spectra of LCHF and DCHF were determined (Fig. S27 and S28†) and exhibited perfect mirror imaging profiles.

On account of the same l-type phenylalanine stereocenter within the LPF and LCHF components and achiral bipyridines used, it was reasonably inferred that the changes of CD signals and unexpected enantiomerically enriched helical nanostructures observed from the SEM images could be attributed to specific stacking modes of co-assembling building blocks, which bring vital effects upon chiroptical behavior and chiral morphology of hydrogels by strong and extensive intermolecular hydrogen bonding between l-phenylalanine derivatives and achiral bipyridines.

### Co-assembly mechanism of hydrogels

**Fig. 4:**
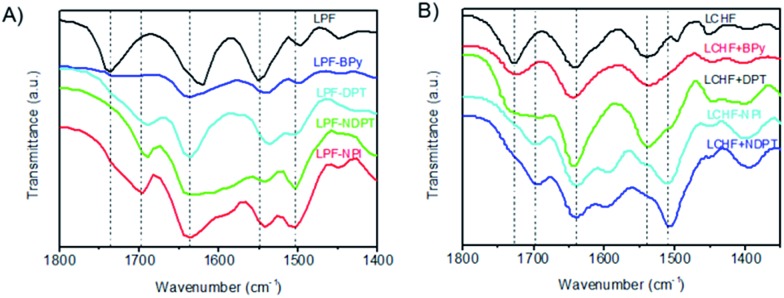
FTIR spectra of xerogels self- or co-assembled from LPF (A) and LCHF (B).

The co-assembling mechanism of these hydrogels was further investigated by Fourier transform infrared spectroscopy (FTIR), since these measurements (Fig. 4 and S29–S35†) can provide valuable information about the interaction of supramolecular aggregates at a molecular level. The as-prepared xerogel of LPF was first characterized by FTIR and showed well-defined amide I bands centered at 1621 cm^–1^, amide II bands centered at 1551 cm^–1^, and stretching vibration bands of C=O from carboxyl groups at 1738 cm^–1^ (Fig. 4 and S31†). These bands shifted in homogeneous dichloromethane solution (Fig. S31 and Table S1†). These observations suggest that well-developed hydrogen bonding networks were formed through the amide and carboxylic acid units in the self-assembled nanofibers. The FTIR spectrum (Fig. S32†) of LPF + BPy gels clearly displayed well-defined amide I and II bands centered at 1636 cm^–1^ and 1543 cm^–1^, respectively, indicating that the amide groups participated in strong hydrogen bonds. On comparing with LPF xerogel, new bands were observed at 2453 and 1951 cm^–1^ and the band at 1713 cm^–1^ clearly decreased, which suggested the formation of carboxylic acid–pyridyl hydrogen bonds in LPT + BPy. Moreover, the appearance of a peak due to the N–H stretching vibration at 3308 cm^–1^ further demonstrated the complicated nature of the hydrogen bonds. Similarly, FTIR spectra of the LPF + DPT, LPF + NPI, and LPF + NDPT xerogels all showed well-defined amide I and II bands centered around 1635 and 1540 cm^–1^, respectively, and the stretching vibration bands of C=O from the carboxylic groups at 1735 cm^–1^ disappeared, coupled with a new peak at ∼1695 cm^–1^. In addition, two peaks assigned to the O–H stretching vibrations at 2495 and 1950 cm^–1^ were observed. FTIR studies confirmed that these co-assembled hydrogel frameworks are stabilized by intermolecular hydrogen bonding interactions between the amide/pyridine units and carboxylic acid groups.

For LCHF-based hydrogel systems (Fig. S33 and S34†), compared with LCHF gel exhibiting a carboxylic band at 1726 cm^–1^ (*ν*_C=O_ of COOH), the carboxylic band of LCHF + BPy gel was observed at 1722 cm^–1^, and a new peak at 2527 cm^–1^ due to the O–H stretching vibrations was inferred from the newly formed hydrogen bonds between pyridyl and carboxylic acid groups. For xerogels of LCHF + DPT, LCHF + NDPT, and LCHF + NPI, amide I bands shifted to 1691, 1694, and 1697 cm^–1^, respectively. Well-defined amide II bands (*δ*_N–H_ of CONH) at 1537 cm^–1^ in LCHF powder shifted to 1535 cm^–1^ for LCHF + BPy, 1537 cm^–1^ for LCHF + DPT, 1506 cm^–1^ for LCHF + NDPT, and 1510 cm^–1^ for LCHF + NPI. In addition, new peaks due to O–H stretching vibrations were observed at 2546 and 1951 cm^–1^ for LPF + DPT, 2566 and 1945 cm^–1^ for LPF + NDPT, and 2503 and 1953 cm^–1^ for LPF + NPI. Thus, the co-assembled hydrogels based on LCHF were also driven by intermolecular hydrogen bonds between amide/pyridine units and carboxylic acid groups.

On the basis of all the abovementioned results, it can be concluded that the main driving forces for the co-assemblies are two types of intermolecular hydrogen bonds. First, a strong hydrogen bond is formed between pyridyl nitrogen and the hydroxy group of a carboxylic acid, which drives the building blocks to co-assemble in a head-to-tail fashion. Second, three-dimensional fiber networks are obtained through the formation of hydrogen bonds between amide groups. These two kinds of intermolecular hydrogen bonds stabilize the co-assembled hydrogel frameworks.

### VCD activity of the co-assembled hydrogels

**Fig. 5:**
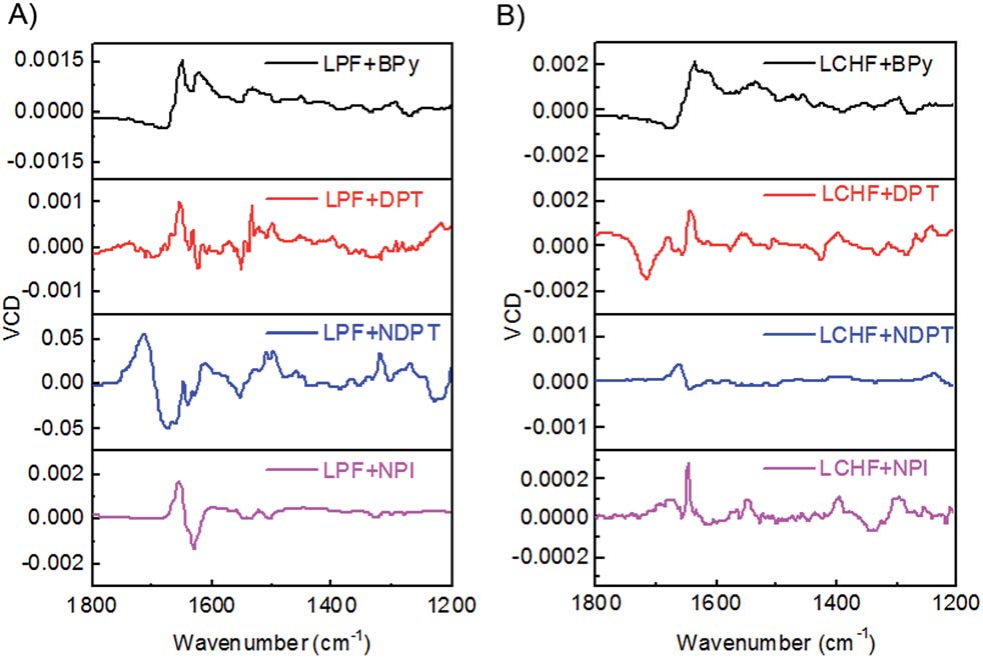
VCD spectra of (A) LPF + BPy, LPF + DPT, LPF + NDPT, and LPF + NPI hydrogels, and (B) LCHF + BPy, LCHF + DPT, LCHF + NDPT, and LCHF + NPI hydrogels.

The chiroptical activities of these hydrogels were also studied by vibrational circular dichroism (VCD).^13^ We conducted the VCD measurements by coating the hydrogels on a CaF_2_ wafer followed by drying under an infrared lamp. For LPF-based hydrogel systems (Fig. 5A), LPF + BPy exhibited a (–/+) VCD signal of C=O stretching band between 1750 and 1600 cm^–1^, whereas the VCD signal of the band switched to a significant (+/–) pattern for LPF + DPT, LPF + NDPT, and LPF + NPI hydrogels. Thus, a strong and extensive C=O···H–N hydrogen-bonding network, significantly stabilizing the co-assembled supramolecular hydrogels, is inferred from the vibrational amide I stretching band at around 1636 cm^–1^. This amide I VCD band in LPF + BPy gives a (–/+) pattern, and that in LPF + DPT, LPF + NDPT, and LPF + NPI showed an opposite (+/–) signal. The VCD patterns imply the inversion of the chirality from LPF + BPy to LPF + DPT/LPF + NDPT/LPF + NPI at room temperature. Surprisingly, all of these VCD bands revealed a (–/+) pattern in the LCHF-based co-assembled hydrogels between 1750 and 1600 cm^–1^, which may be ascribed to the different central cyclohexyl unit in LCHF (Fig. 5B).

Since all samples have the same S-type stereocenter within the l-phenylalanine units, dissimilar VCD behavior between LPF + BPy gel and LPF + DPT/LPF + NDPT/LPF + NPI gels suggested that their supramolecular self-assembly could result in the formation of distinct aggregates with opposite handedness using different achiral counterparts. The present results already indicate that LPF can assemble into different enantiomerically enriched helical supramolecular structures with achiral bipyridines. In this very rare two-component supramolecular hydrogel system, unexpected right-handed nanostructures (except for LPF + BPy) were successfully constructed by the co-assembly of l-phenylalanine derivatives with achiral bipyridines, strongly revealing that the supramolecular chirality of the nanostructures is not only determined by the chirality of monomers (LPF and LCHF), but also highly influenced by the stacking mode of building blocks through strong and extensive intermolecular hydrogen bonding during the co-assembling process. On the basis of these results, it can be confirmed that the intermolecular hydrogen bonding between COOH–pyridine and amide–amide leads to different interaction modes of LPF and LCHF with achiral bipyridines. These two types of intermolecular hydrogen bonding interactions could possibly enable the building blocks to assemble into uniform helical aggregates.

## Conclusions

In conclusion, unexpected right-handed helical nanostructures were successfully constructed by the co-assembly of l-phenylalanine derivatives with achiral bipyridines in a two-component supramolecular approach. In this way, we were able to demonstrate that the chirality of supramolecular architectures could be determined by both the molecular chirality and the stacking mode of the building blocks in the co-assembly process. Studying a series of right-handed helical nanofibers containing building blocks with the same l-type chiral stereocenter and achiral bipyridines with different molecular conformation enabled us to gain an insight into the conveyance of configurational information during helical nanostructure formation. With the generality of this approach demonstrated for a number of different building block combinations, we expect that this approach should be applicable to a broad variety of building blocks for promoting the establishment of chiral nanomaterials with desirable topologies. Further investigations using this strategy to define chiral relationship between enantiomeric monomers and supramolecular assemblies would bring new insights into the deeper understanding of the chiral assembly process and regulation of the supramolecular aggregation.

## Experimental

### General

The NMR spectra were obtained using a Bruker Advance III 300 Instrument (300 MHz). HRMS were determined using a Water Q-Tof Mass Instrument. Amino-4-pyridine, 1,4-benzene-dicarbonyl dichloride, 4,4′-bipyridine, 4-carboxylic pyridine, 1,4-cyclohexane dicarboxylic acid, 4-dimethylaminopyridine (DMAP), 1-ethyl-3-(3-dimethylamino propyl)carbodiimide hydrochloride (EDCI), 4-hydroxypyridine, l-phenylalaninemethylester hydrochloride, thionyl chloride, and triethylamine (Et_3_N) were purchased from Aladdin Chemicals.

### Synthesis of LCHF

1,4-Cyclohexanedicarboxylic acid (1.73 g, 10.00 mmol) was added to dry dichloromethane containing thionyl chloride (20 mL), and the mixture was stirred at 100 °C for 4 h. All the solvents were evaporated under vacuum and the residue liquid was collected to give 1,4-cyclohexanedicarbonyl dichloride. 1,4-Cyclohexane dicarbonyldichloride (2.0 g, 9.66 mmol) in dry dichloromethane (100 mL) was added dropwise to a dichloromethane solution (100 mL) containing l-phenylalaninemethyl ester hydrochloride (5.0 g, 23.18 mmol) and triethylamine (3.6 mL, 26.00 mmol) in an ice-water bath. After completing the addition, the solution was stirred at room temperature overnight. All the solvents were evaporated under vacuum and the residue was subsequently dissolved in dichloromethane (100 mL). After washing with water, the organic phase was dried by anhydrous MgSO_4_ and collected to give the dimethyl ester of LCHF (LCHF-OMe, 4.60 g, 9.30 mmol, 84%). ^1^H NMR (300 MHz, DMSO-*d*_6_, ppm): *δ* = 1.43 (t, 4H, CH_2_), 1.86 (m, 4H, CH_2_), 2.05 (s, 2H, CH), 3.11 (dd, 4H, CH_2_), 3.71 (s, 6H, CH_3_), 4.87 (d, 2H, CH), 5.92 (d, 2H, CO–NH), 7.07 (m, 4H, Ar-H), 7.27 (d, 6H, Ar-H). ^13^C NMR (101 MHz, DMSO-*d*_6_, ppm): *δ* = 174.96, 172.34, 136.03, 128.76, 127.36, 125.47, 52.96, 52.54, 44.49, 38.04, 28.78, 28.49.

For the hydrolysis, aqueous NaOH (10 mL, 2.0 M) was added to a cooled suspension of LCHF-OMe (5.43 g, 6.14 mmol) in MeOH (20 mL). The mixture was slowly heated to room temperature and stirred for 24 h, and a clear solution was obtained. The solution was then acidified with 3.0 M HCl until the pH value was not more than 3.0, and gel-like precipitate was formed. The gel phase was filtered, washed with deionized water, and finally dried in the vacuum oven to give LCHF (3.0 g, 6.38 mmol, 69.2%). Overall yield of LCHF: 66.6%. ^1^H NMR (300 MHz, DMSO-*d*_6_, ppm): *δ* = 12.66 (s, 2H, COOH), 8.06 (d, 2H, CONH), 7.30 (m, 10H, Ar-H), 4.45 (s, 2H, CH), 2.98 (m, 4H, CH_2_), 2.10 (s, 2H, CH), 1.63 (d, 4H, CH_2_), 1.24 (d, 4H, CH_2_). ^13^C NMR (75 MHz, DMSO-*d*_6_, ppm): *δ* = 174.31, 172.70, 137.52, 128.90, 127.89, 126.16, 53.42, 43.33, 37.20, 28.66. EI-MS for C_26_H_30_O_6_N_2_ calcd 466.2104; found 467.2180 [M + H]^+^.

### Synthesis of DPT

1,4-Benzenedicarbonyl dichloride (1.01 g, 4.98 mmol) in dry dichloromethane (10 mL) was added dropwise to a dichloromethane solution (20 mL) containing 4-hydroxypyridine (1.42 g, 14.93 mmol), EDCI (2.24 g, 11.94 mmol), and DMAP (0.06 g, 0.50 mmol). The solution was stirred at room temperature for 12 h. After filtration, all the solvents were evaporated under vacuum. The residue was washed with deionized water, and finally dried in a vacuum oven to give solid DPT (0.96 g, 3.00 mmol, 60.2%). ^1^H NMR (400 MHz, DMSO-*d*_6_, ppm): *δ* = 8.71–8.72 (d, 4H, Ar-H), 8.35 (s, 4H, Ar-H), 7.26–7.29 (dd, 4H, Ar-H). ^13^C NMR (100 MHz, DMSO-*d*_6_, ppm): *δ* = 176.77, 167.36, 140.24, 135.28, 129.82, 116.49. EI-MS (*m*/*z*) for C_18_H_14_N_2_O_4_ calcd 320.0797; found 321.0869 [M + H]^+^.

### Synthesis of NPI

4-Carboxylicpyridine (0.61 g, 4.95 mmol) in dry dichloromethane (10 mL) was added dropwise to a dichloromethane solution (20 mL) containing amino-4-pyridine (0.75 g, 7.97 mmol), EDCI (0.99 g, 5.16 mmol) and DMAP (0.04 g, 0.33 mmol). The solution was stirred at room temperature for 12 h. After filtration, all the solvents were evaporated under vacuum. The residue was washed with deionized water, and finally dried in a vacuum oven to obtain claybank solid NPI (0.23 g, 1.15 mmol, 23.3%). ^1^H NMR (400 MHz, DMSO-*d*_6_, ppm): *δ* = 7.78–7.80 (d, 2H, Ar-H), 7.87–7.89 (d, 2H, Ar-H), 8.52–8.53 (d, 2H, Ar-H), 8.82–8.84 (d, 2H, Ar-H), 10.84 (s, 1H, CO–NH). ^13^C NMR (100 MHz, DMSO-*d*_6_, ppm): *δ* = 166.64, 150.70–150.92, 147.60, 143.60, 122.93, 115.65. EI-MS (*m*/*z*) for C_11_H_9_N_3_O calcd 199.0746; found 200.0811 [M + H]^+^.

### Hydrogel preparation

The LPF + DPT hydrogel with 0.2 wt% LPF + DPT is used as an example to describe the preparation procedure. LPF + DPT (2.0 mg mL^–1^, an equimolar mixture of LPF and DPT) was suspended in a septum-capped 5.0 mL glass vial and heated until a homogeneous solution was obtained. The solution solidified into a hydrogel after standing for a half-hour at room temperature.

### Scanning electron microscopy (SEM)

SEM was performed using a JEOL JSM-7600F microscope with an accelerating voltage of 5 kV. Before performing the SEM measurements, the samples were prepared by depositing dilute solutions of gels on silicon wafers, followed by drying and coating them with a thin layer of Pt to increase the contrast.

### Circular dichroism (CD) spectra

CD spectra were obtained using JASCO J-1500 CD spectrometer with bandwidth of 1.0 nm. CD spectra of the hydrogels were obtained in the UV region (190–400 nm) using a 0.1 mm quartz cuvette with a total gelator concentration at 0.2 wt%.

### Fourier transform infrared (FTIR) spectra

FTIR spectra of the xerogels were acquired using a Shimadzu FT-IR Instrument. The KBr disk technique was used for the solid-state measurements. Solution spectra were obtained by dropping dichloromethane solution onto KBr wafers and were corrected for solvent and cell absorption. The samples were scanned between 4000 and 400 cm^–1^ wavelengths at an interval of 1.9285 cm^–1^.

### Vibrational circular dichroism (VCD) spectra

VCD spectra were obtained by BioTools using a ChiralIR-2X Fourier transform VCD (FT-VCD) spectrometer equipped with an MCT detector and the Dual PEM option for enhanced VCD baseline stability. VCD spectra were acquired at a resolution of 4 cm^–1^ by co-adding 1000 scans. The gel samples (at a concentration of 2.0 mg mL^–1^) were dried under infrared lamp after coating on a CaF_2_ wafer that is subsequently placed in a variable path length cell with CaF_2_ windows.

## Supplementary Material

Supplementary information
