# Supplementary material for: Unexpected right-handed helical nanostructures co-assembled from l-phenylalanine derivatives and achiral bipyridines
Source: Chem Sci. 2017 Jan 4;8(3):1769–75. doi: 10.1039/c6sc04808k (PMC5933425; doi:10.1039/c6sc04808k)
Supplement: Supplementary file 1 [file SC-008-C6SC04808K-s001.pdf]

Electronic Supplementary Material (ESI) for Chemical Science.
This journal is © The Royal Society of Chemistry 2017

Electronic Supplementary Information (ESI)

Unexpected Right-Handed Helical Nanostructures Co-Assembled from L-Phenylalanine Derivatives and Achiral Bipyridines

Guofeng Liu,[a,b] Jinying Liu,[a] Chuanliang Feng,*[a] and Yanli Zhao*[b,c]

[a]State Key Lab of Metal Matrix Composites, School of Materials Science and Engineering, Shanghai Jiao Tong University, 800 Dongchuan Road, Shanghai, 200240, China

[b]Division of Chemistry and Biological Chemistry, School of Physical and Mathematical Sciences, Nanyang Technological University, 21 Nanyang Link, 637371, Singapore

[c]School of Materials Science and Engineering, Nanyang Technological University, 50 Nanyang Avenue, 639798, Singapore

E-mail: clfeng@sjtu.edu.cn; zhaoyanli@ntu.edu.sg

Additional experimental data and figures

1 NMR spectra of LCHF, DPT, NDPT, and NPI

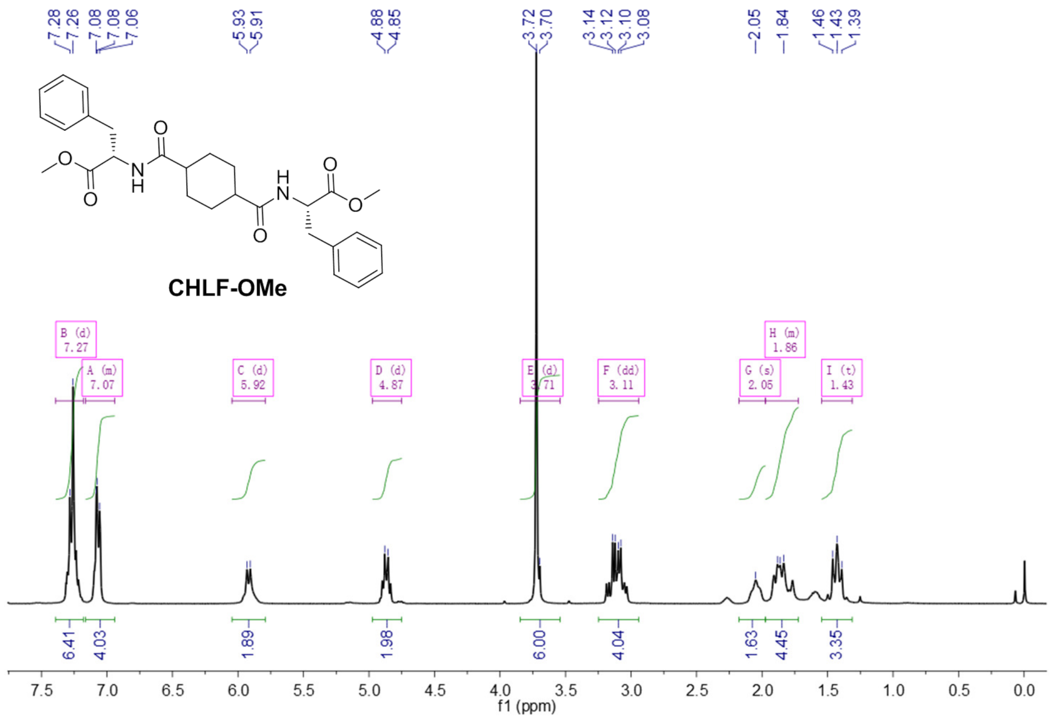

Figure S1. ^{1}H NMR spectrum of LCHF-OMe in $CDCl_3$.

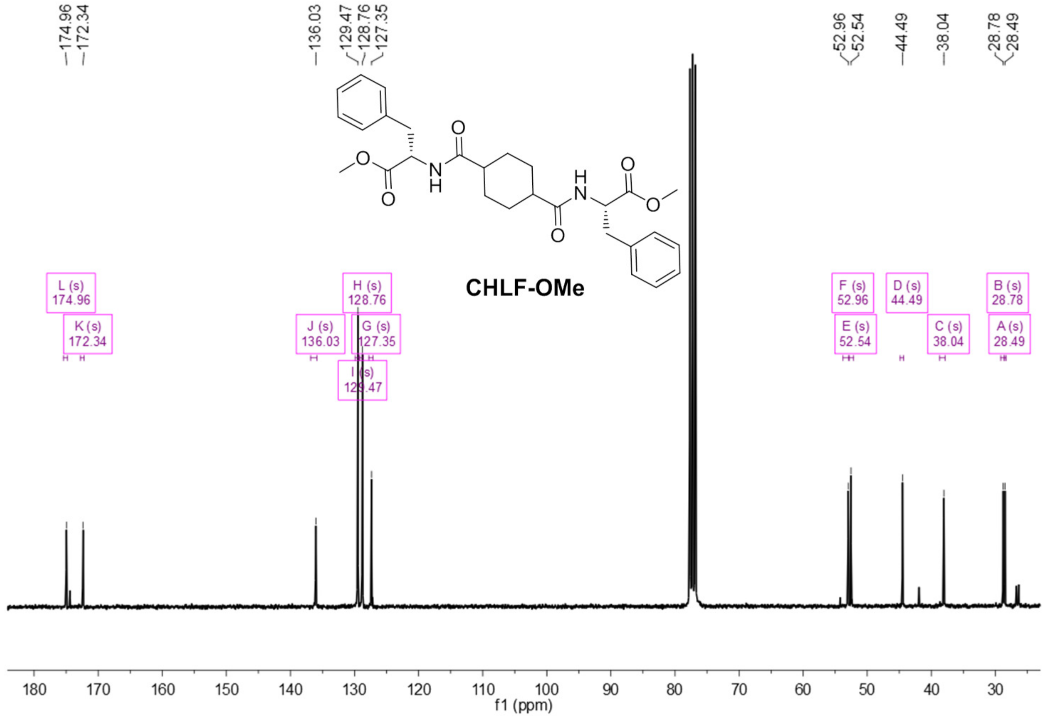

Figure S2. ^{13}C NMR spectrum of LCHF-OMe in $CDCl_3$.

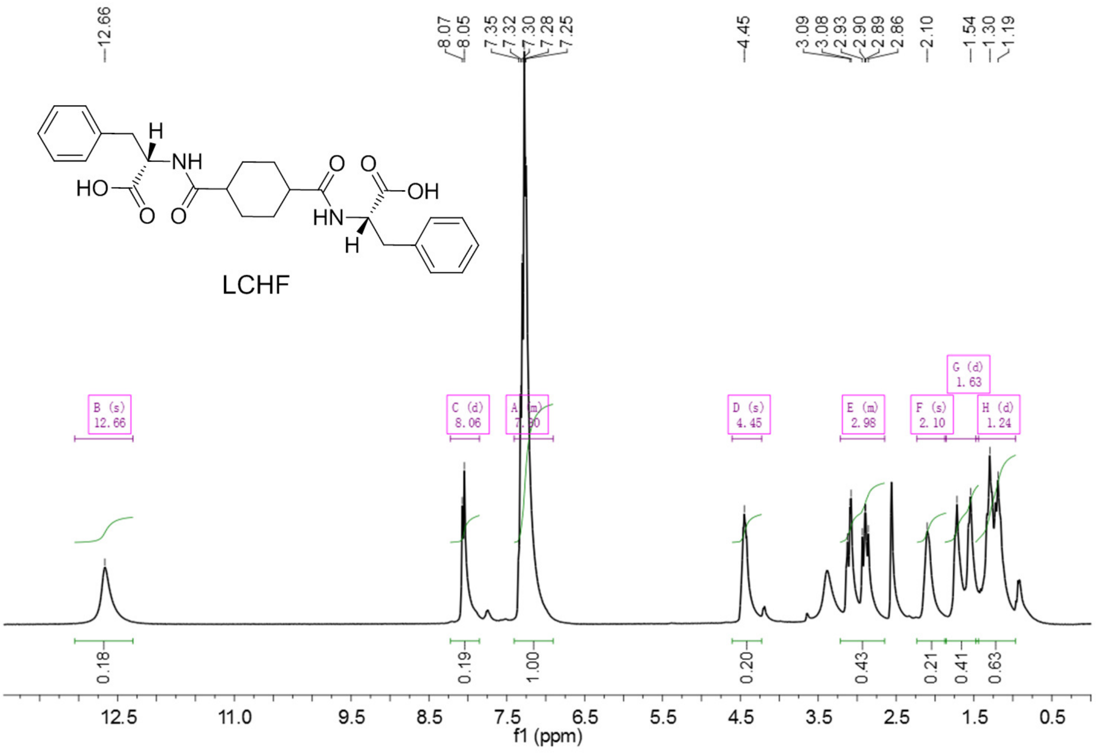

Figure S3. ^{1}H NMR spectrum of LCHF in DMSO-d_6.

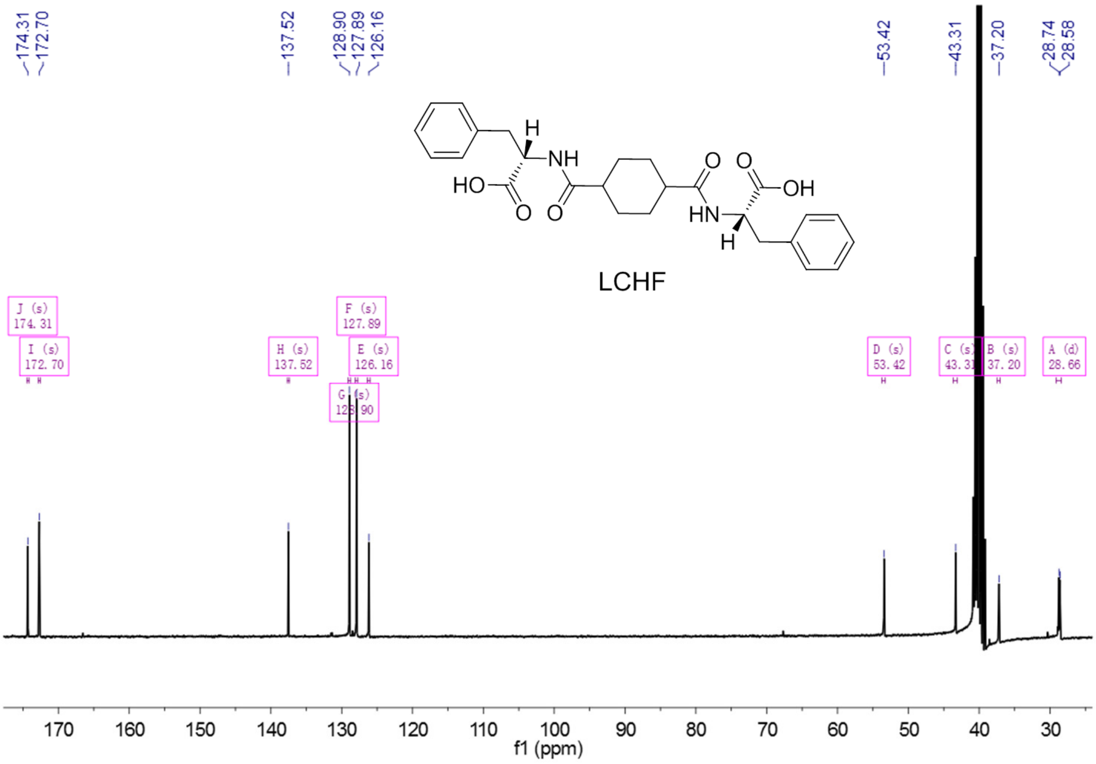

Figure S4. ^{13}C NMR spectrum of LCHF in DMSO-d_6.

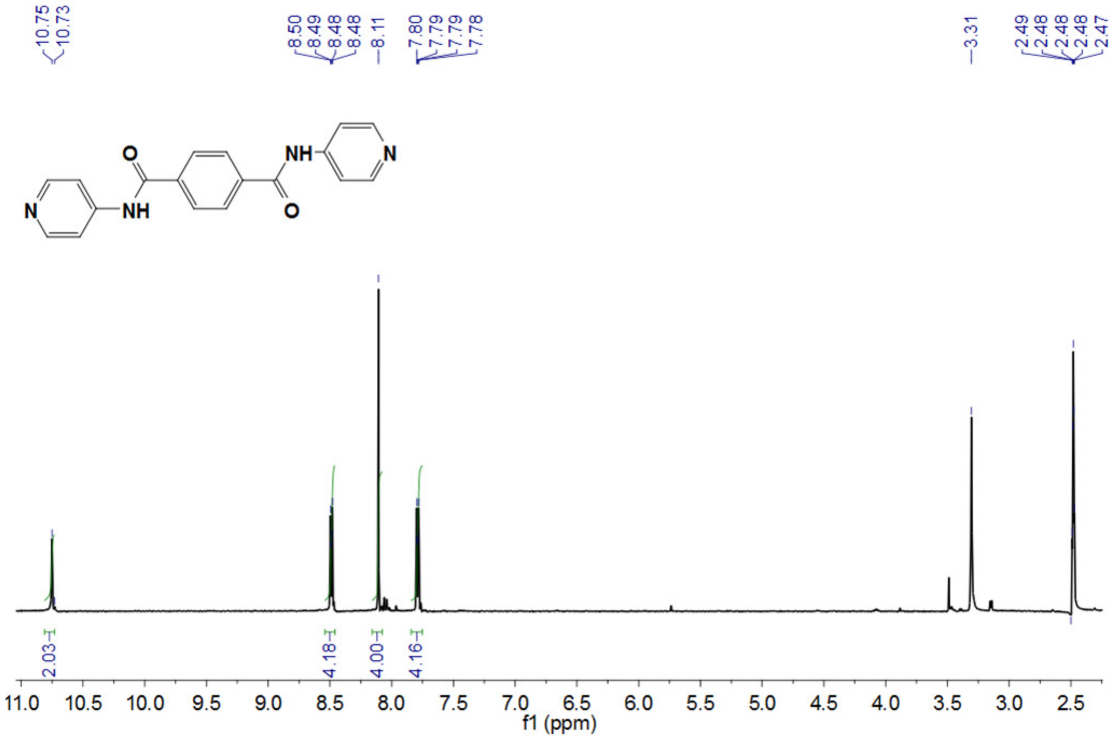

Figure S5. ^{1}H NMR spectrum of NDPT in DMSO-d_6.

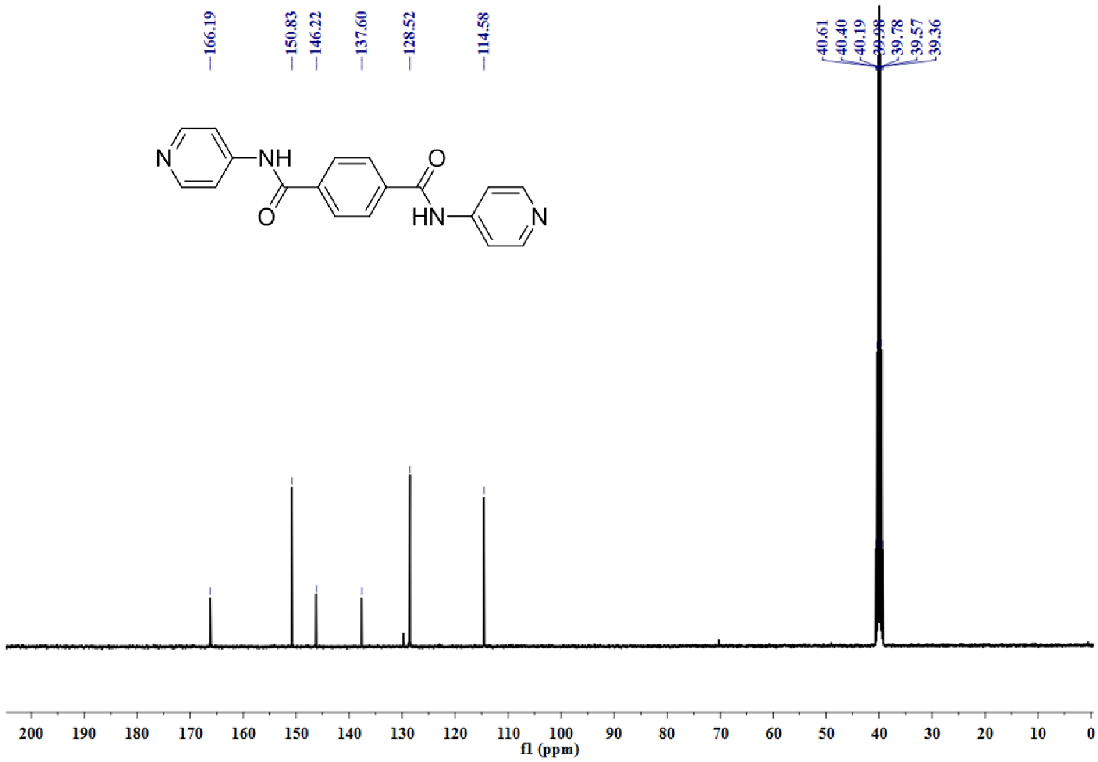

Figure S6. ^{13}C NMR spectrum of NDPT in DMSO-d_6.

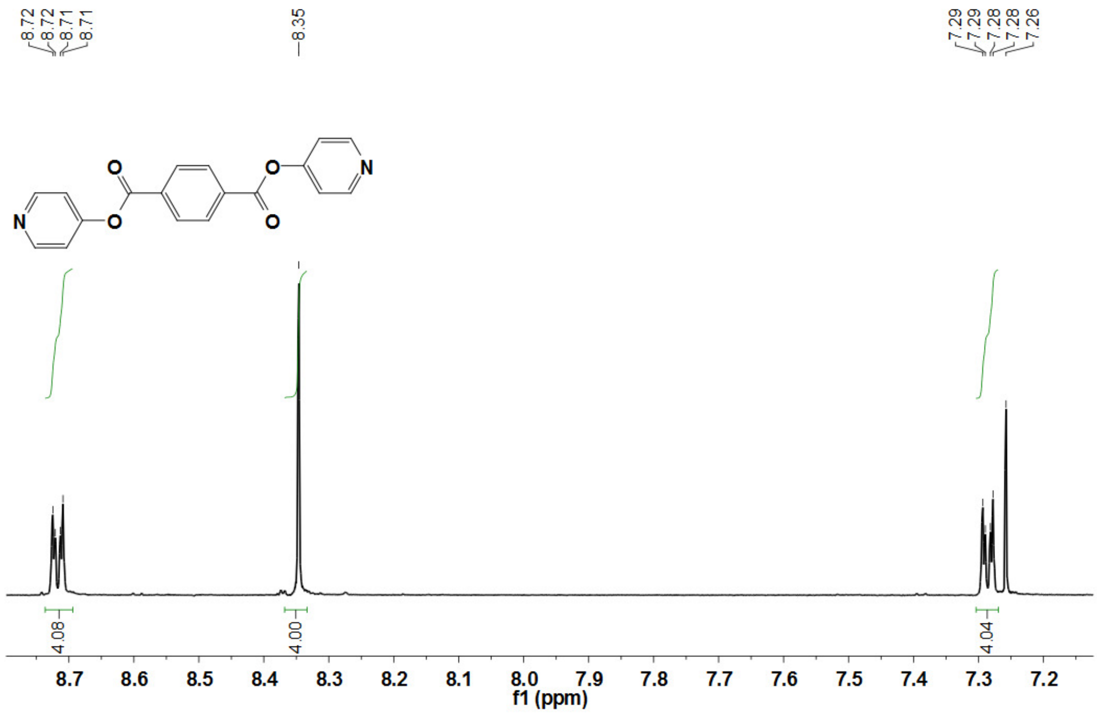

Figure S7. ^{1}H NMR spectrum of DPT in $CDCl_3$.

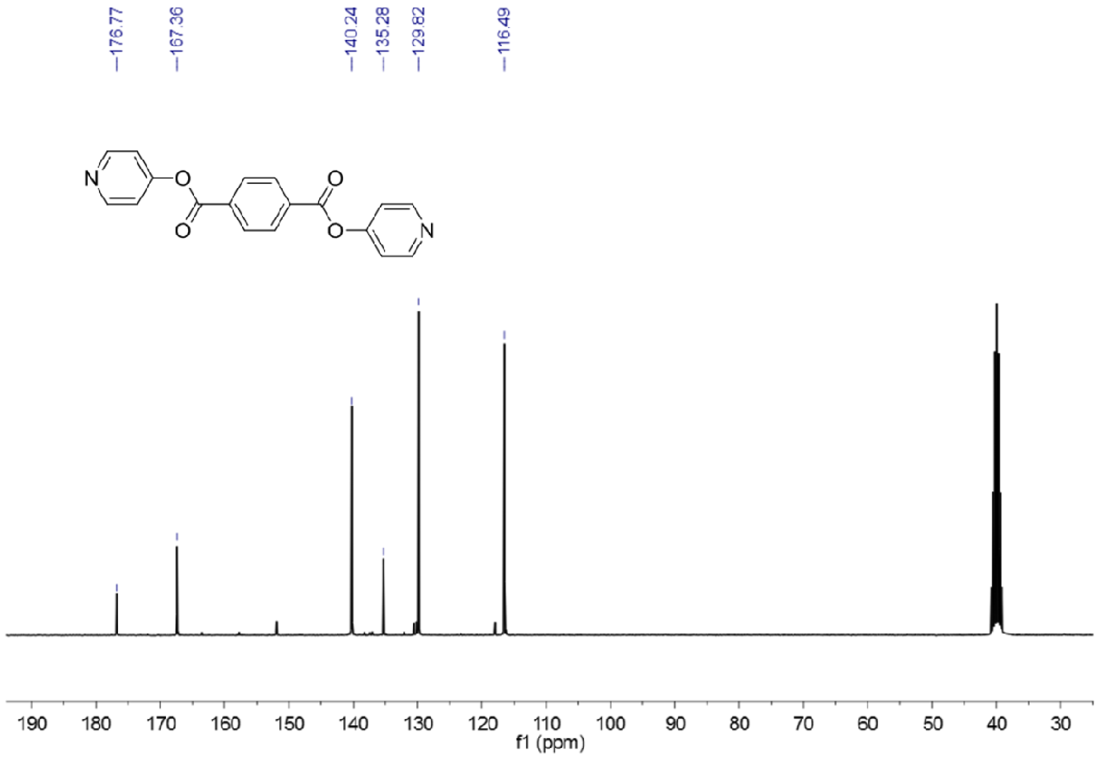

Figure S8. ^{13}C NMR spectrum of DPT in DMSO-d_6.

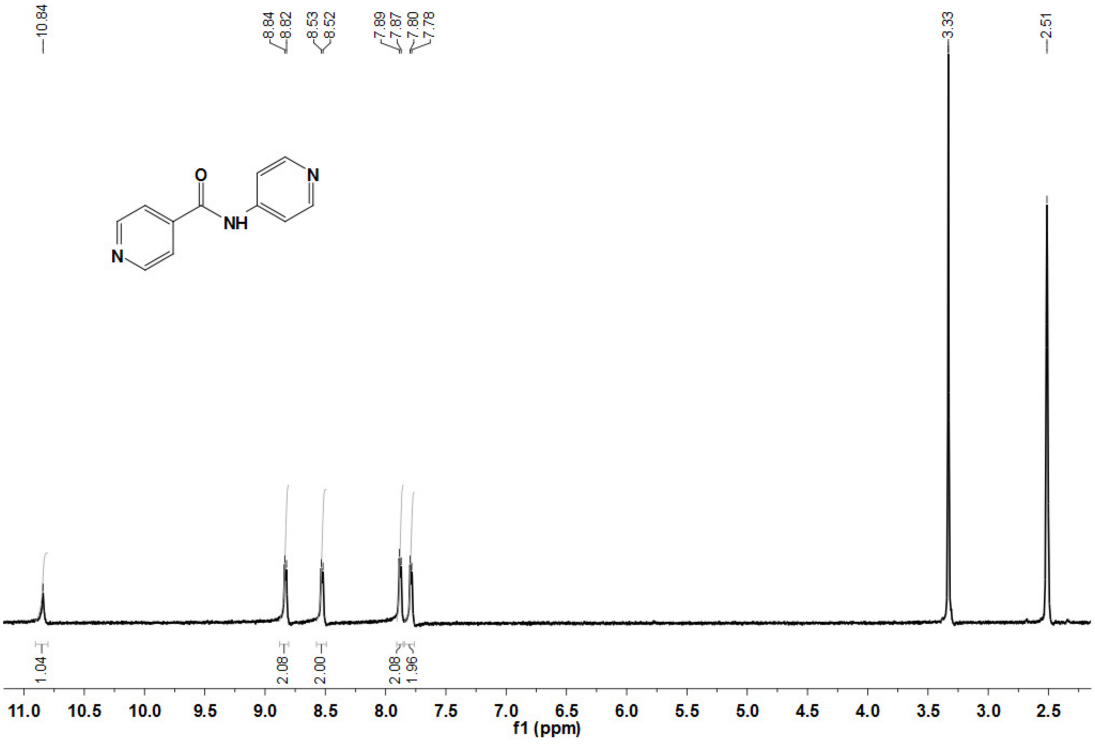

Figure S9. ^{1}H NMR spectrum of NPI in DMSO-d_6.

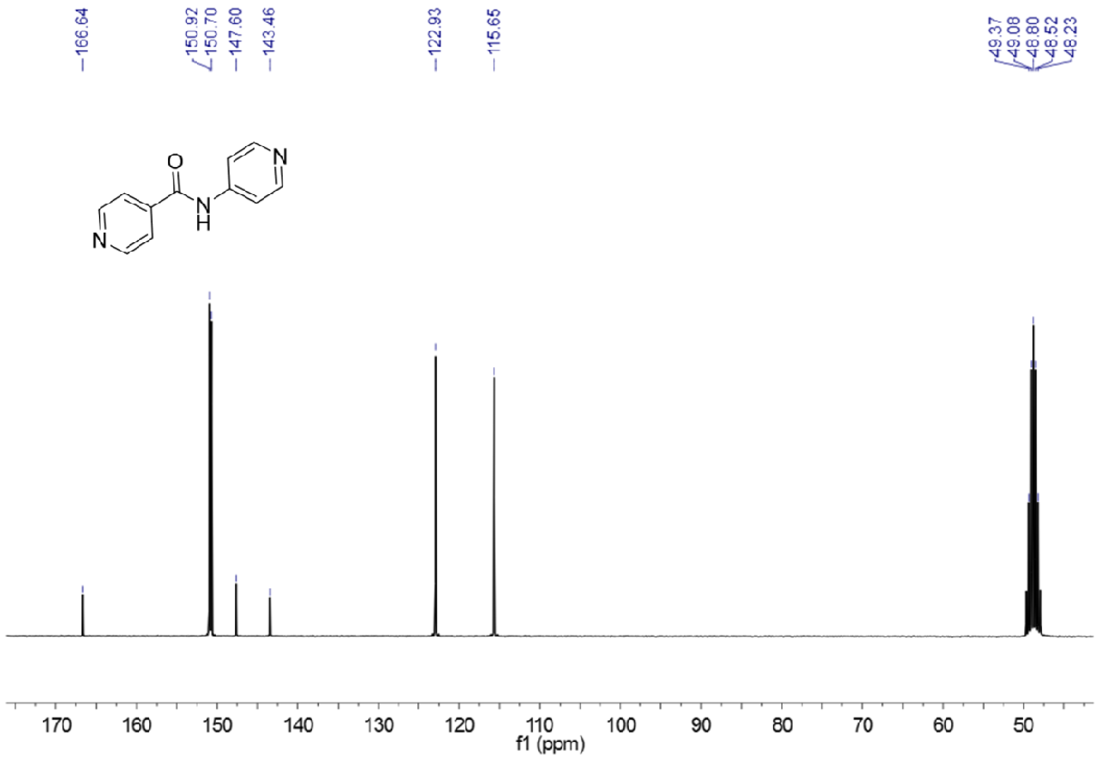

Figure S10. ^{13}C NMR spectrum of NPI in DMSO-d_6.

2 HRMS spectra of LCHF, DPT, NDPT, and NPI

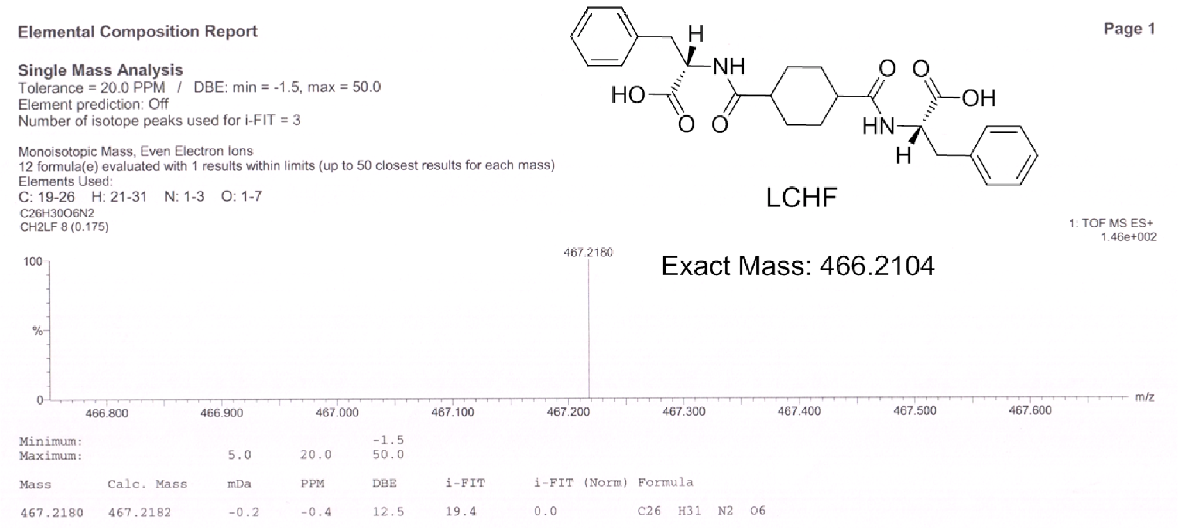

Figure S11. HRMS spectrum of LCHF in methanol.

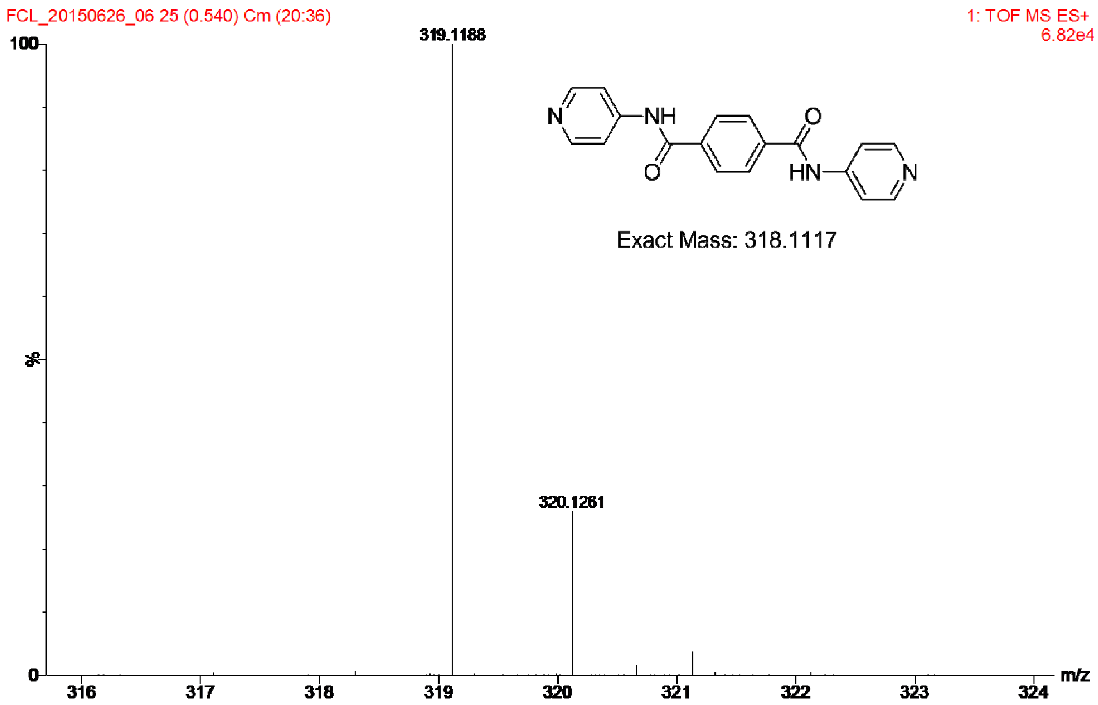

Figure S12. HRMS spectrum of NDPT in methanol.

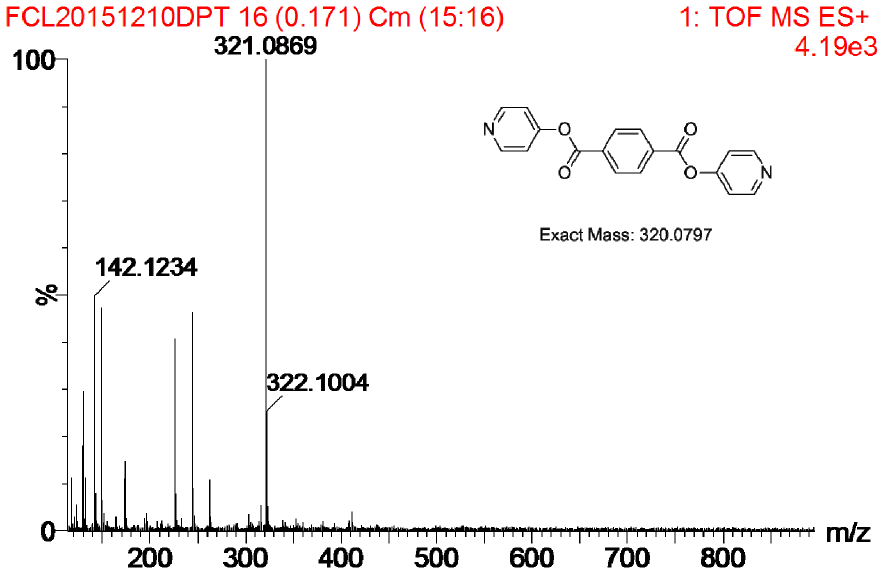

Figure S13. HRMS spectrum of DPT in methanol.

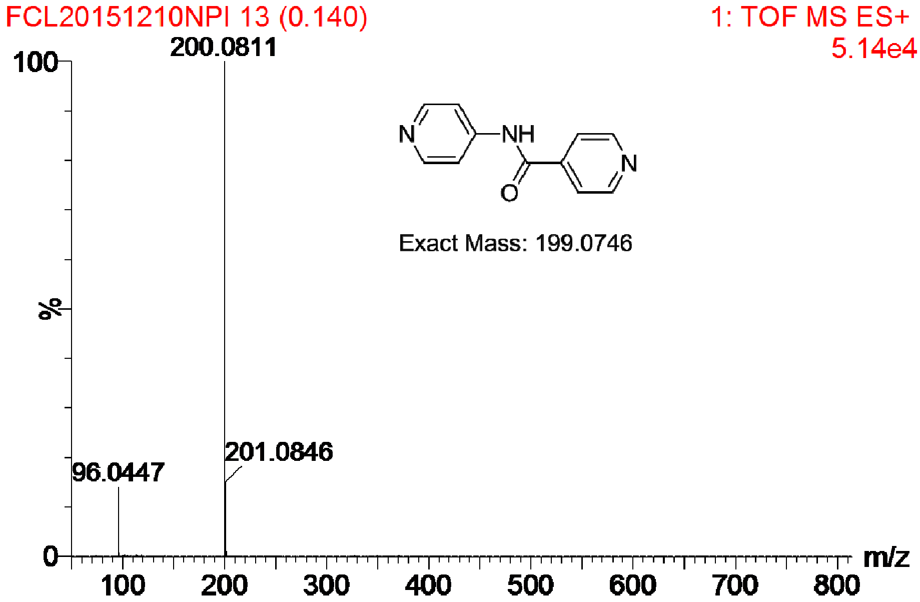

Figure S14. HRMS spectrum of NPI in methanol.

3 Images of hydrogels

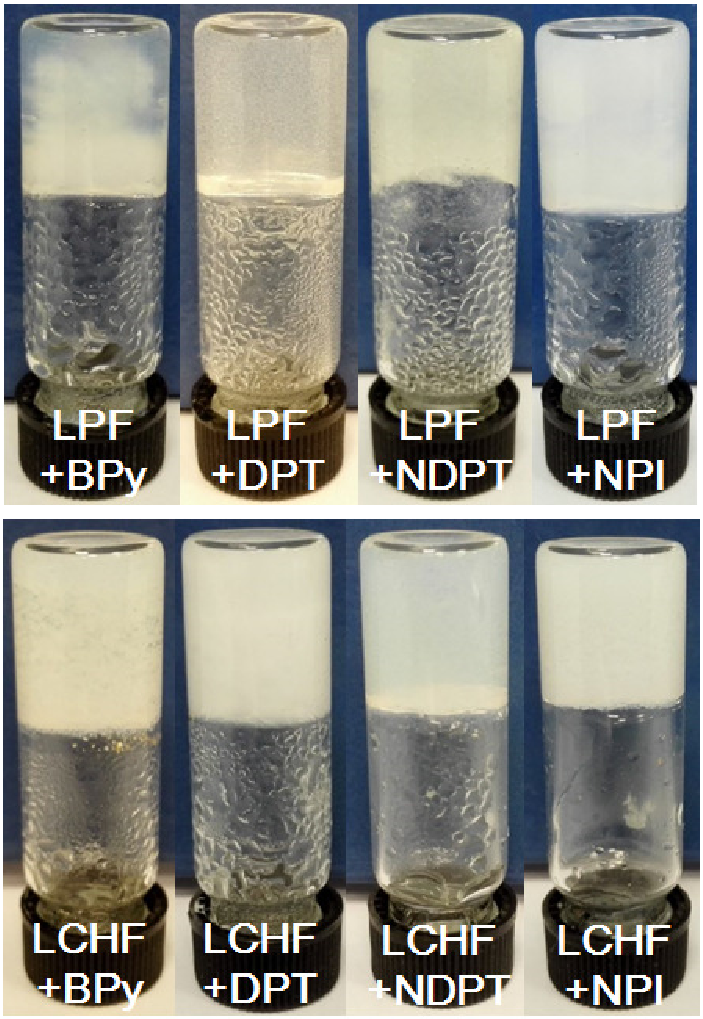

Figure S15. Photographs of co-assembled hydrogels of LPF/LCHF+BPy, LPF/LCHF+DPT, LPF/LCHF+NDPT and LPF/LCHF+NPI prepared at 2.0 mg/mL.

4 SEM images of hydrogels

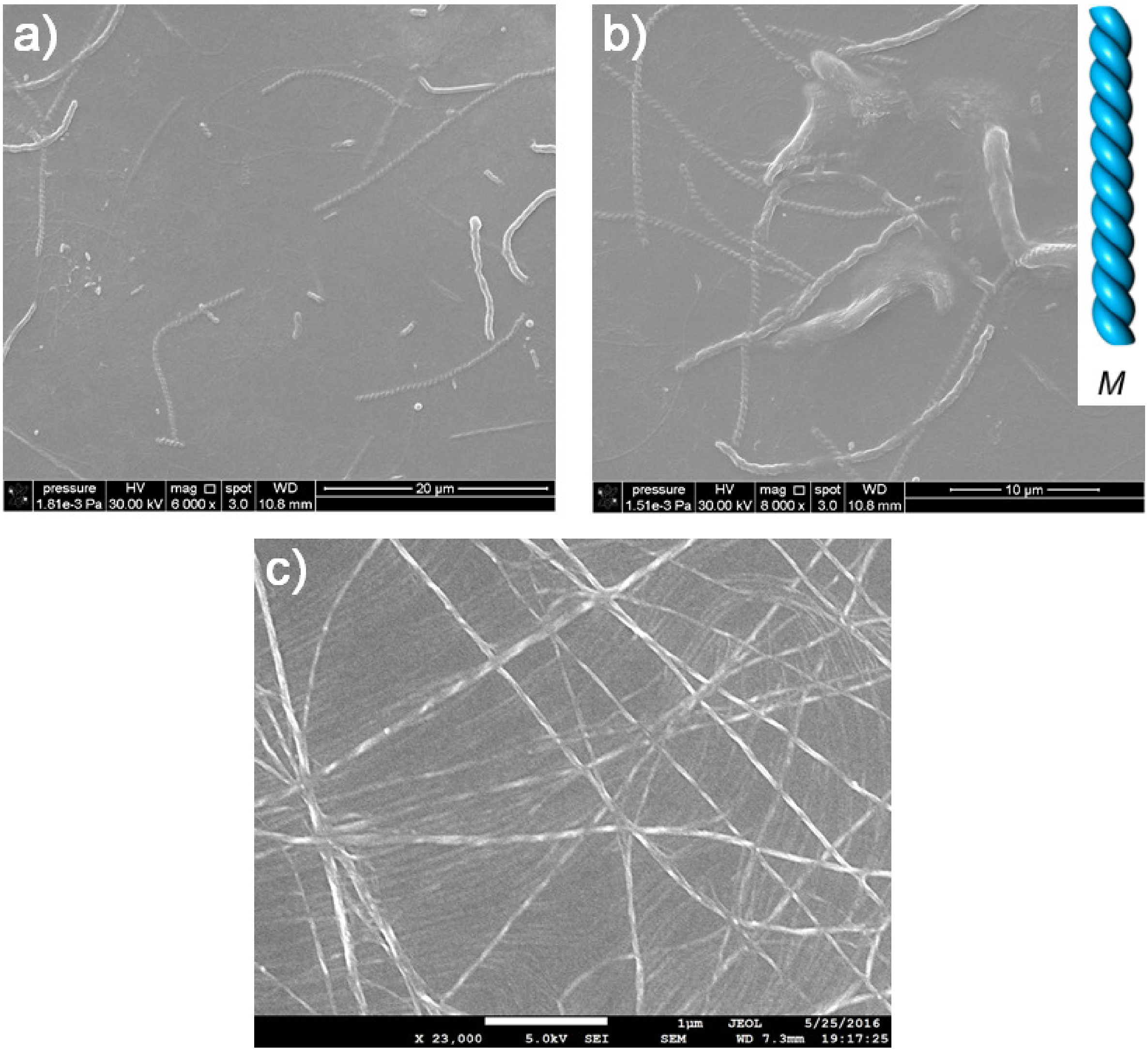

Figure S16. SEM images of co-assembled hydrogel LPF+BPy.

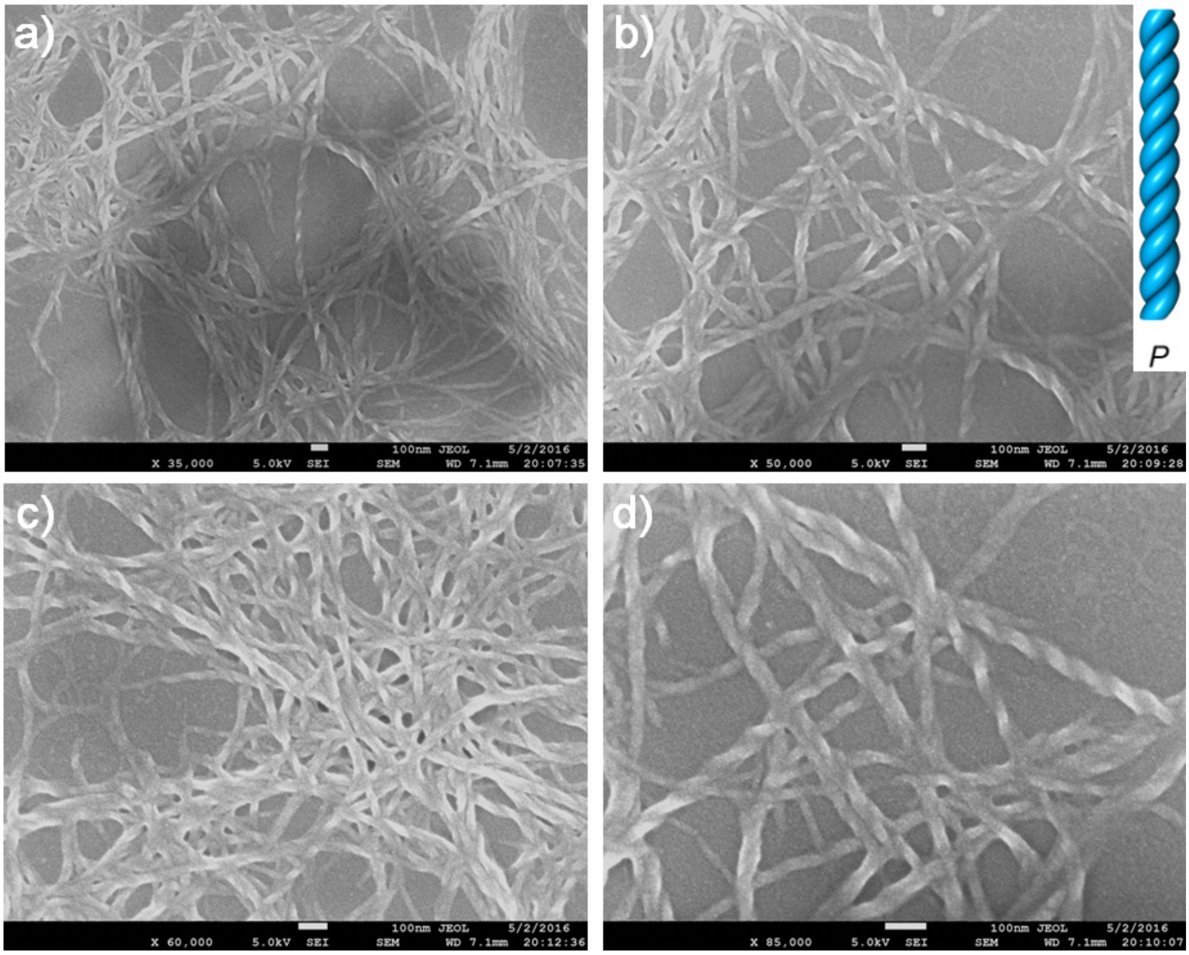

Figure S17. SEM images of co-assembled hydrogel LPF+DPT.

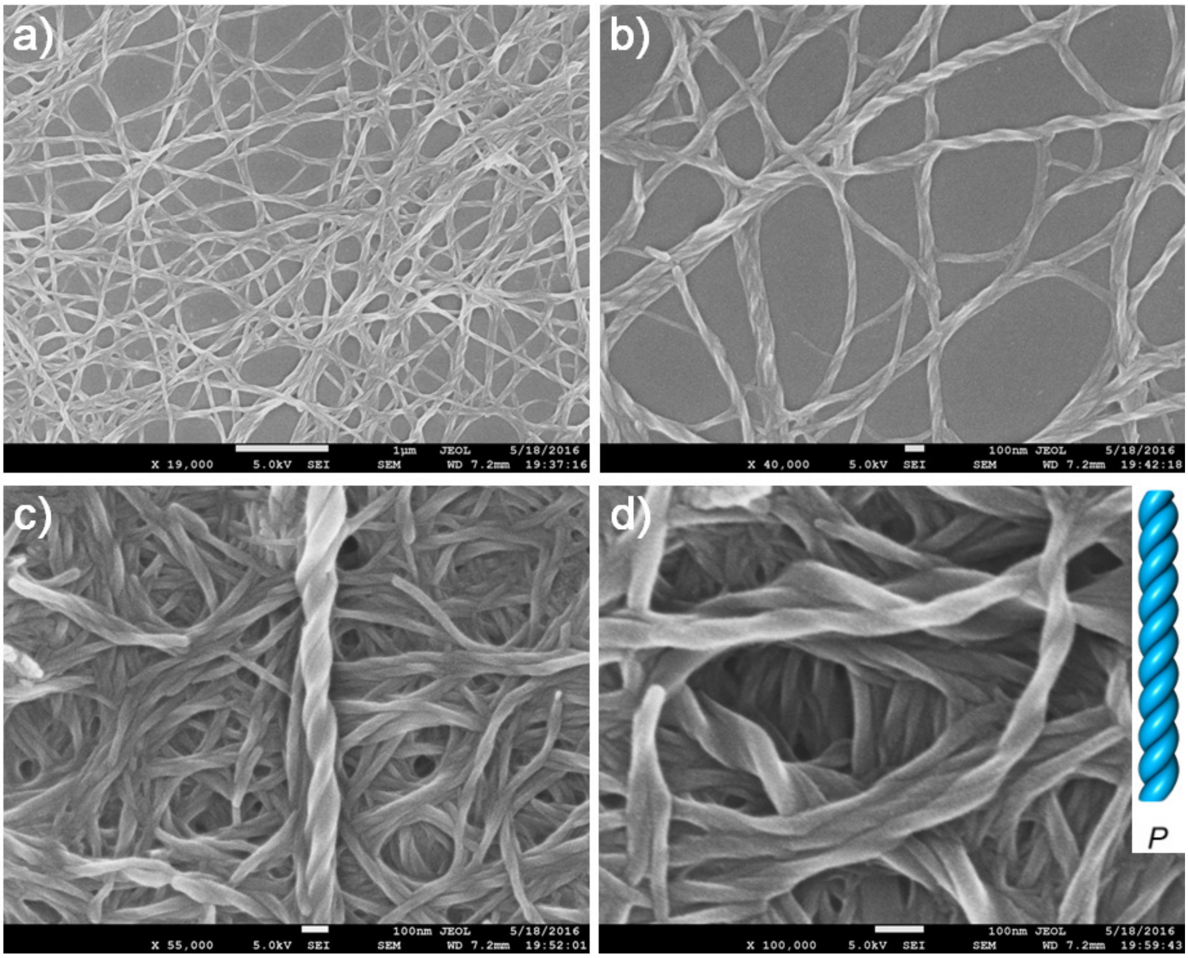

Figure S18. SEM images of co-assembled hydrogel LPF+NDPT.

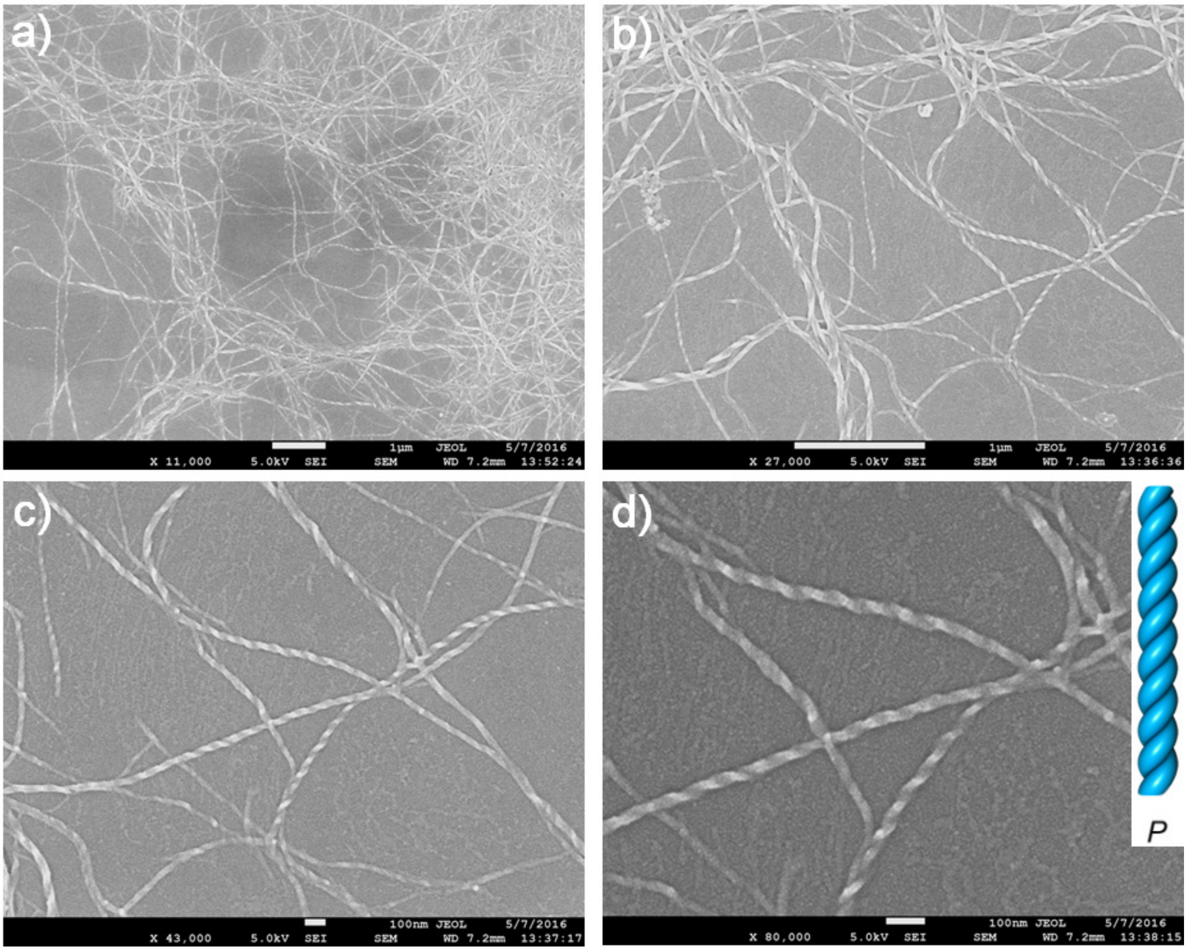

Figure S19. SEM images of co-assembled hydrogel LPF+NDPT.

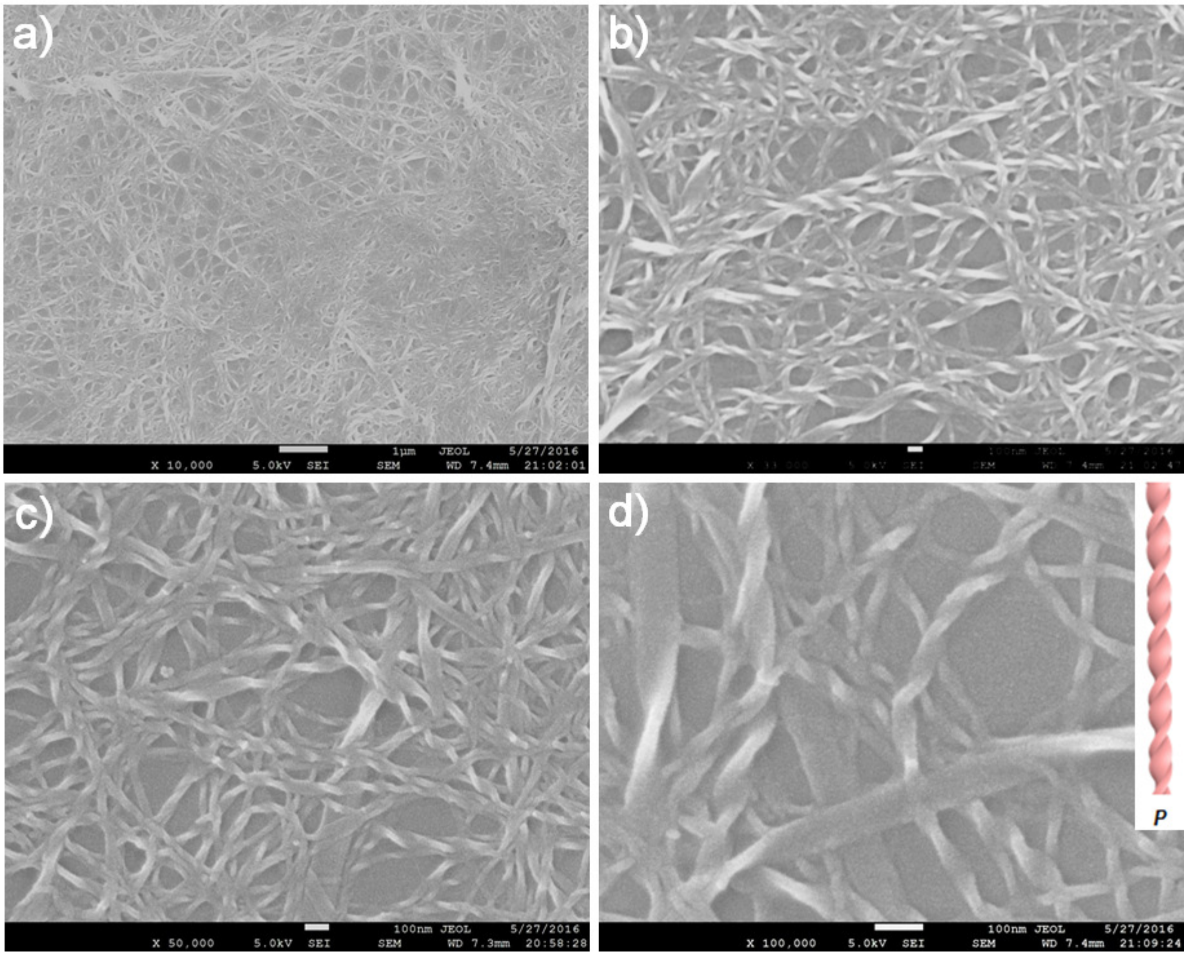

Figure S20. SEM images of co-assembled hydrogel LCHF+BPy.

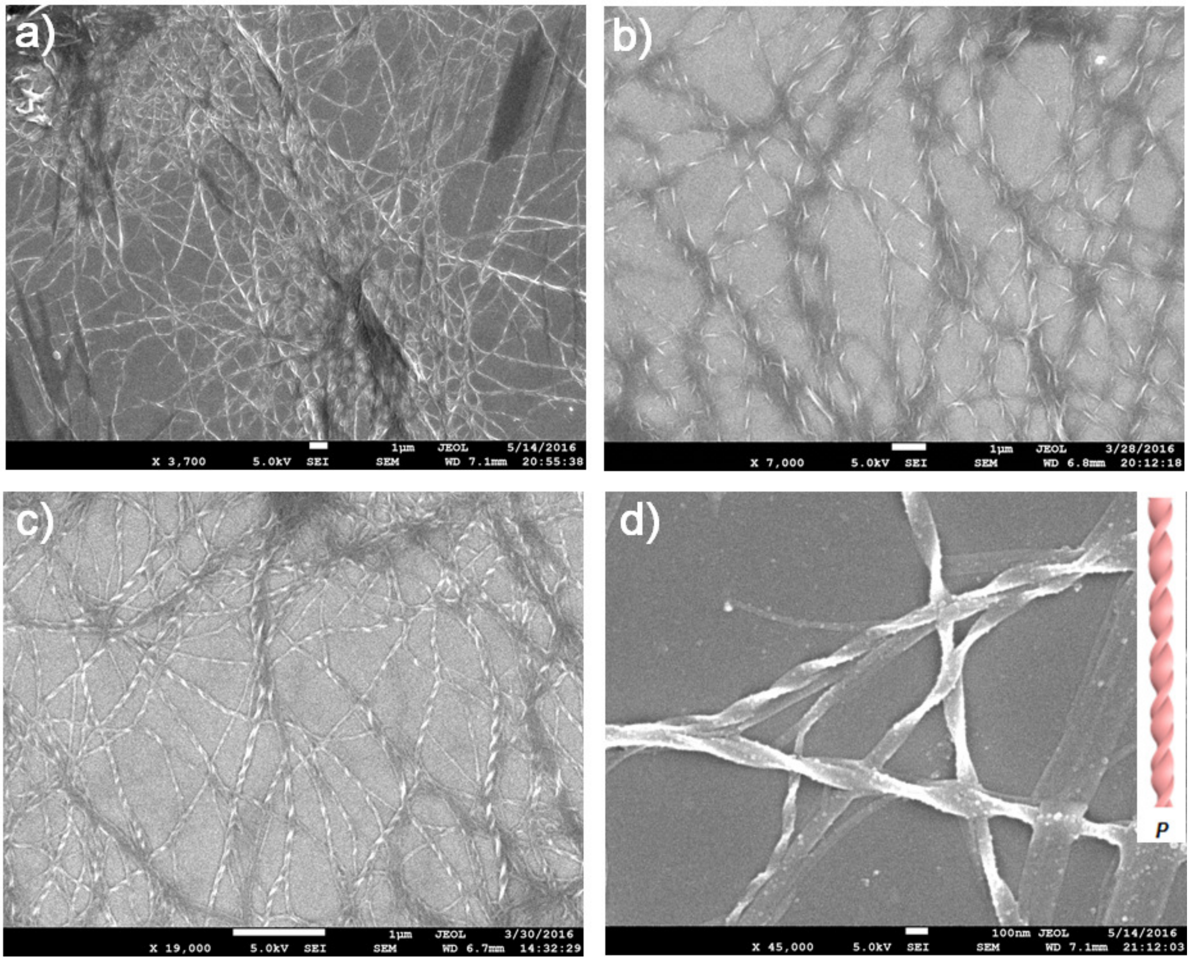

Figure S21. SEM images of co-assembled hydrogel LCHF+DPT.

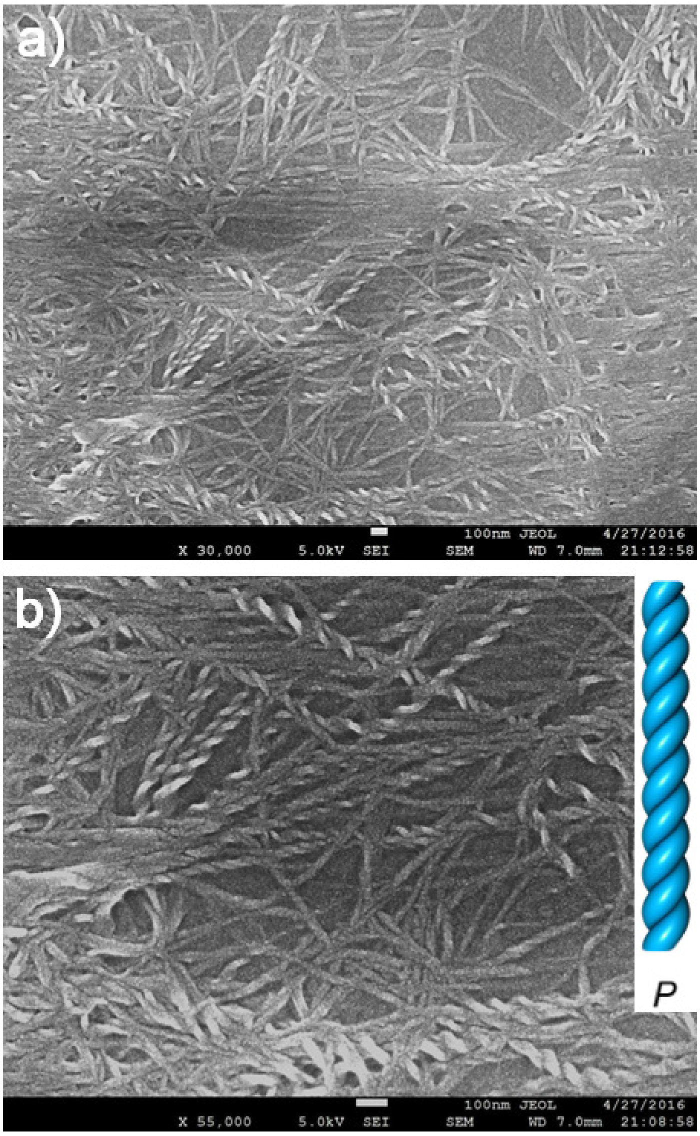

Figure S22. SEM images of co-assembled hydrogel LCHF+NDPT.

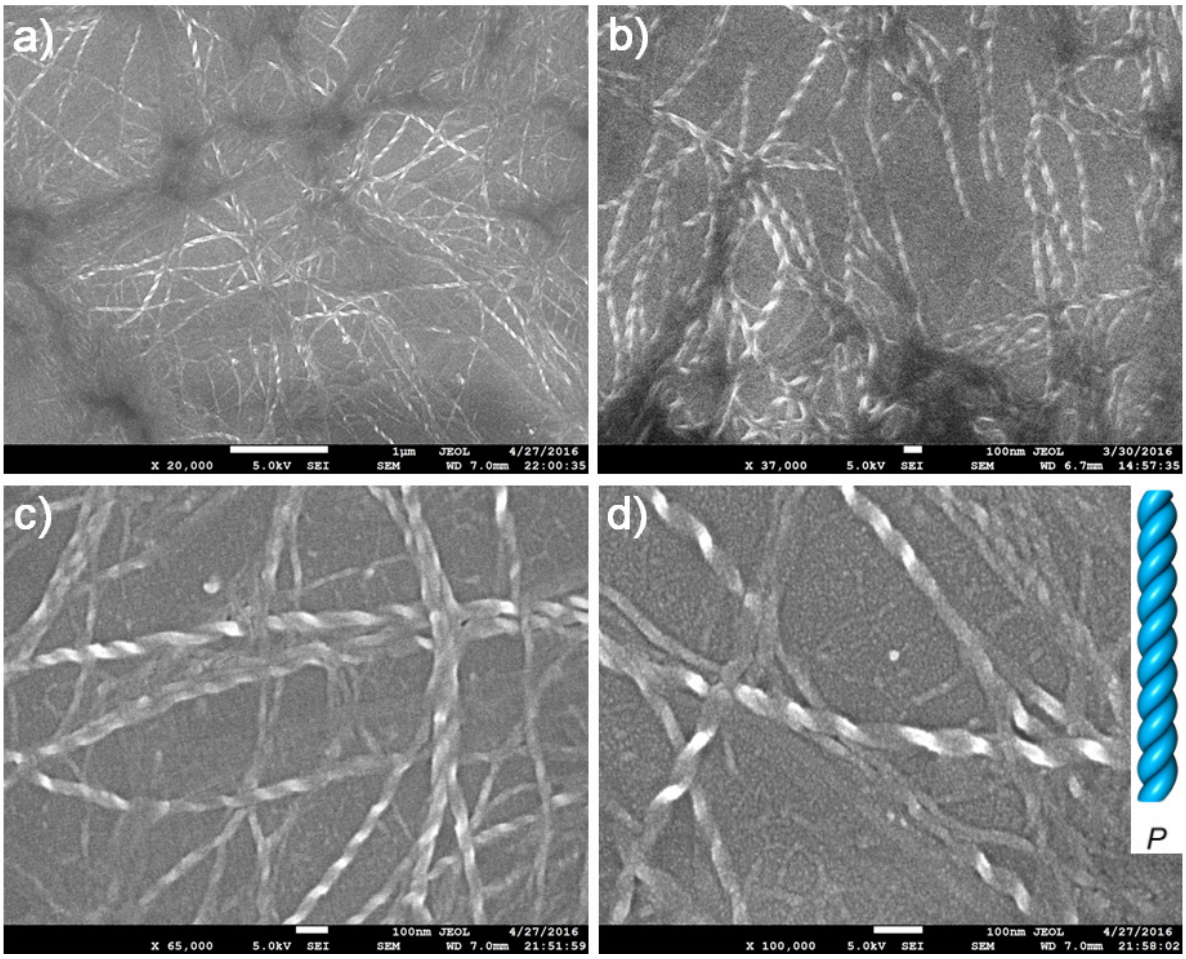

Figure S23. SEM images of co-assembled hydrogel LCHF+NPI.

5 CD, LD and VCD spectra

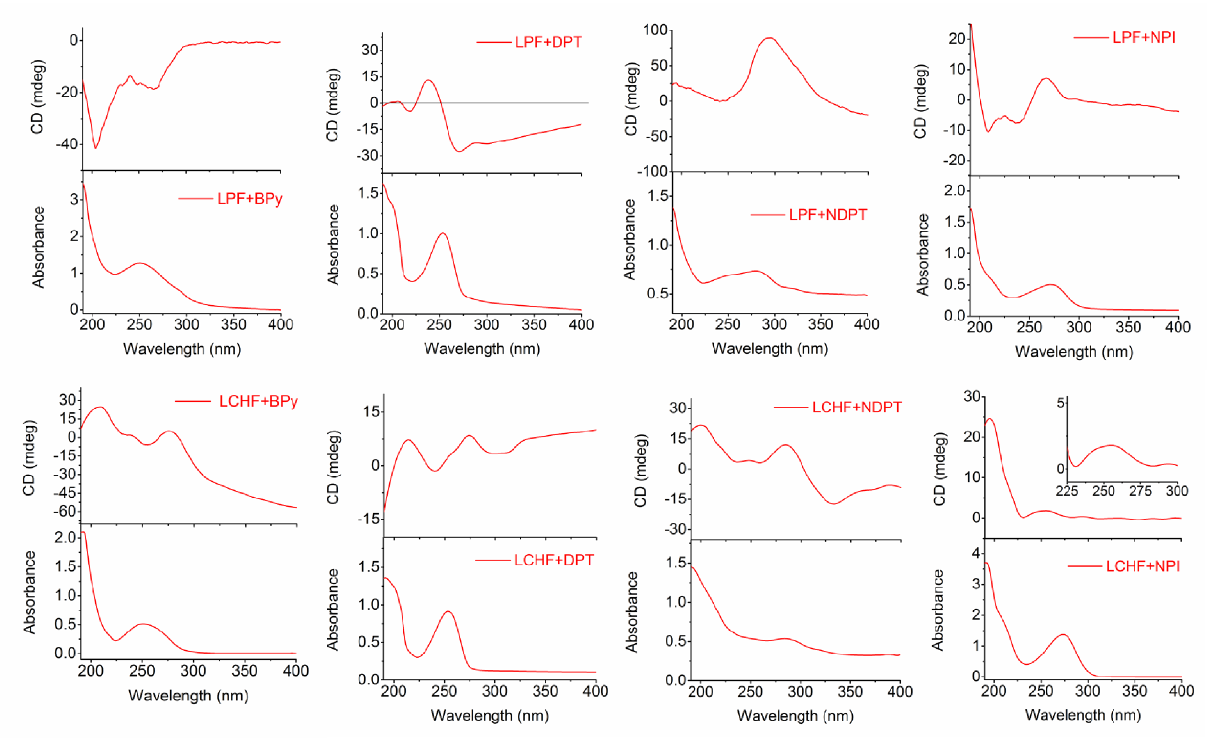

Figure S24. CD and UV-vis spectra of hydrogels based on LPF/LCHF co-assembled with various achiral bipyridines (BPy, DPT, NDPT, and NPI).

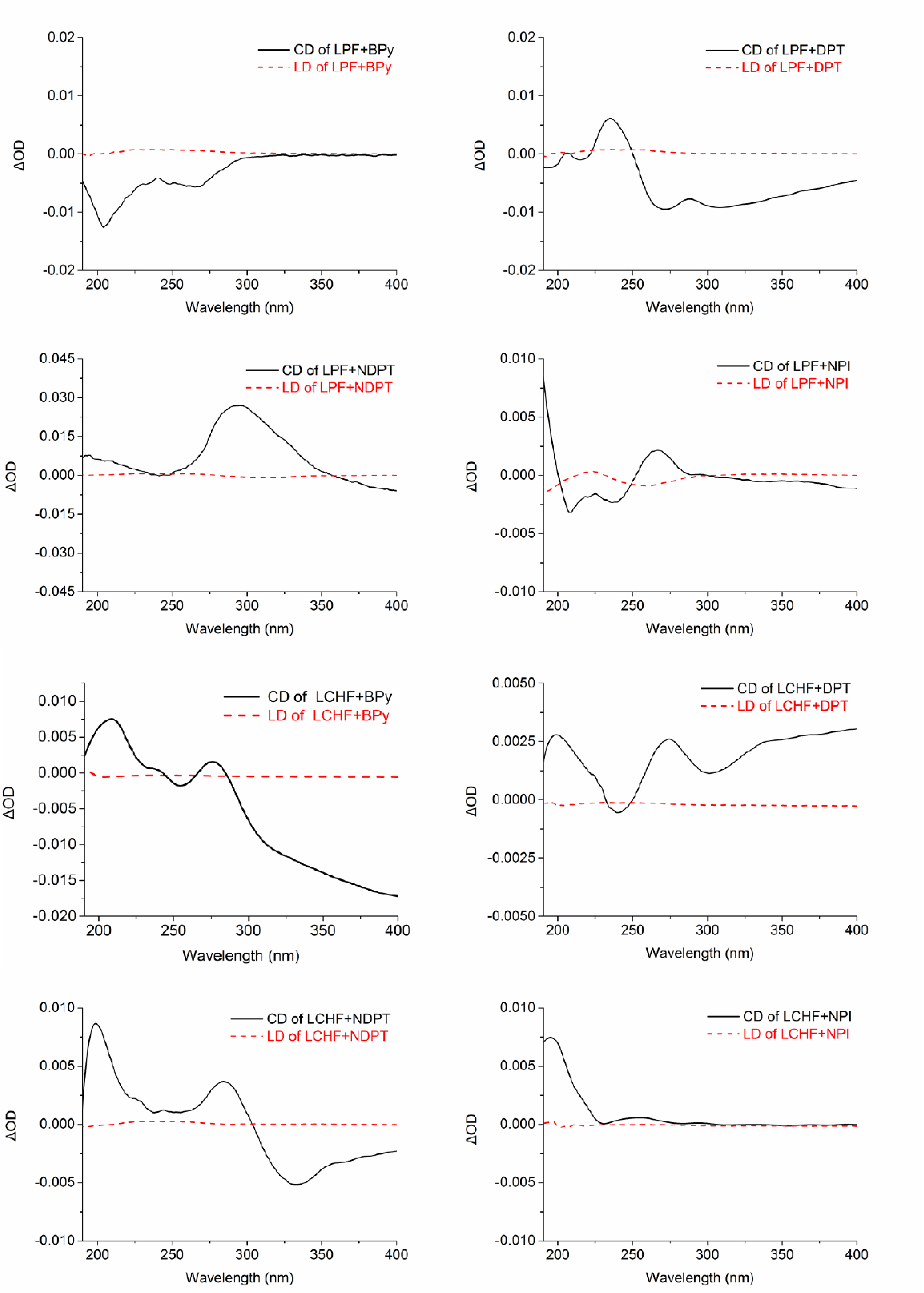

Figure S25. CD and LD spectra of hydrogels based on LPF/LCHF co-assembled with various achiral bipyridines (BPy, DPT, NDPT, and NPI). The solid lines are CD spectra of hydrogels. The dotted lines are LD spectra of the hydrogels. It is obvious that the LD contributions are negligible as compared with that of corresponding CD absorption, except for that of LPF+NPI hydrogel.

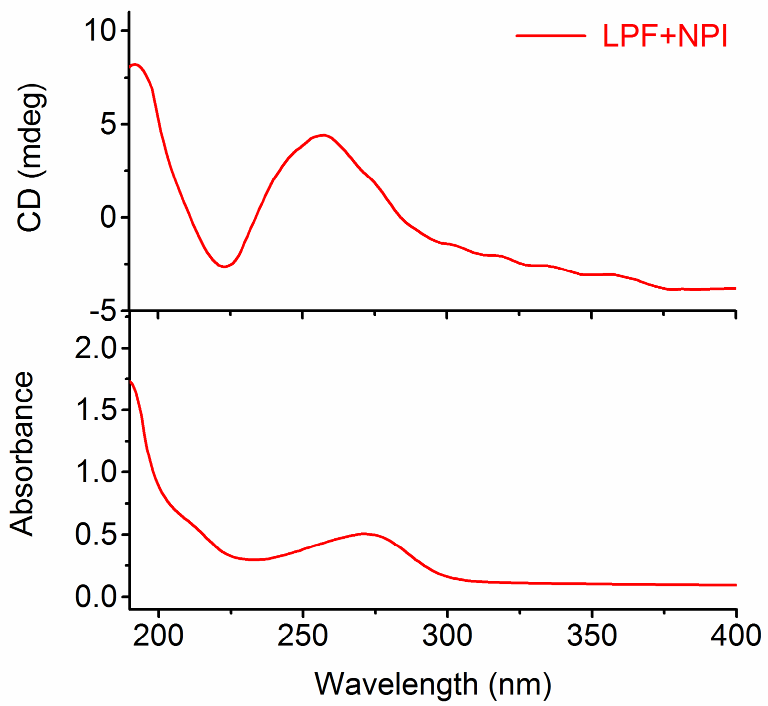

Figure S26. Average CD (upper) and UV-vis (bottom) spectra of LPF+NPI hydrogel. To get rid of the LD influence, the hydrogel film of LPF+NPI was placed in different angles and an average CD signal was taken according to reported procedures in literature.[S1]

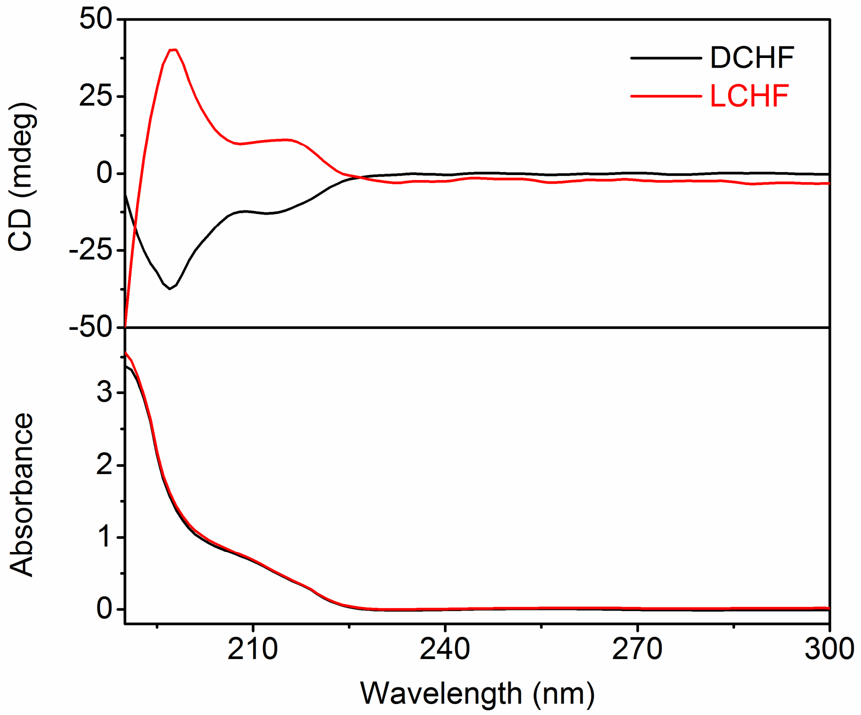

Figure S27. CD (upper) and UV-vis (bottom) spectra of DCHF and LCHF in aqueous solution.

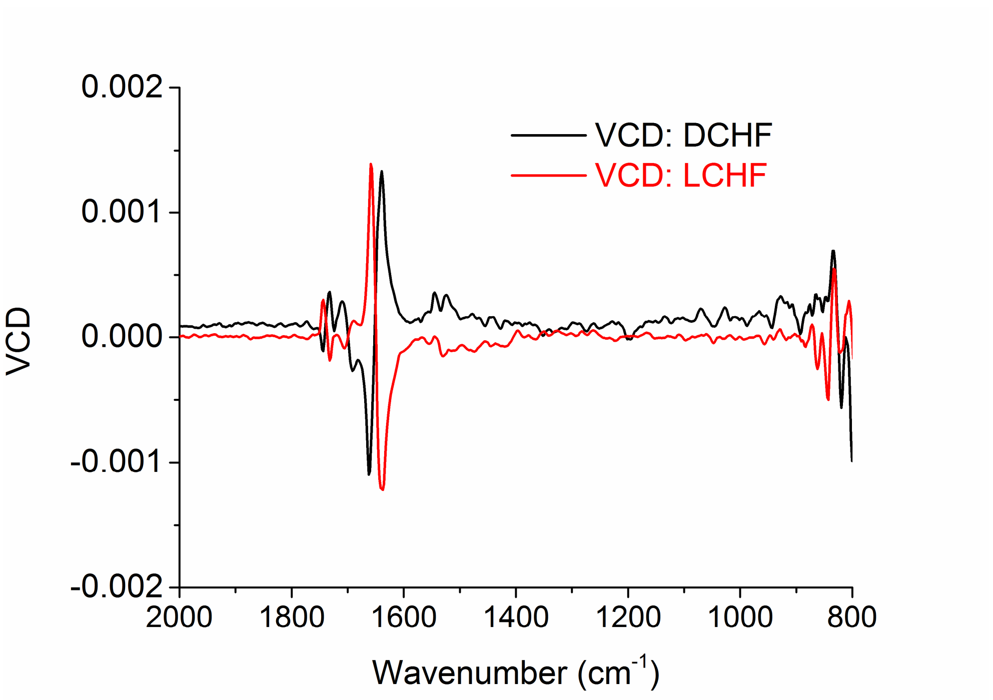

Figure S28. VCD spectra of DCHF and LCHF.

6 FT-IR spectra of xerogels, powders, and solutions in DCM

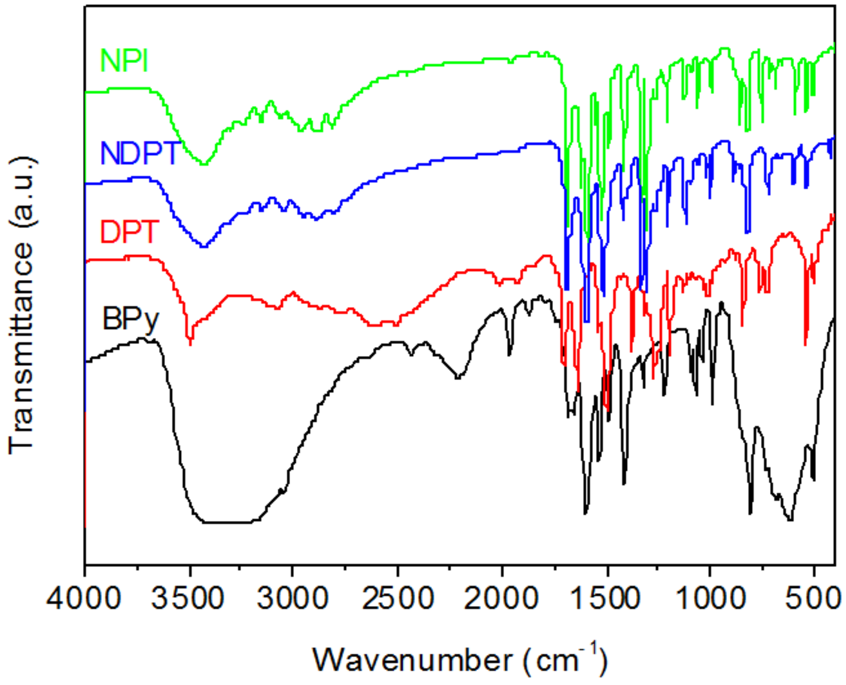

Figure S29. FT-IR spectra of the powder samples for BPy, DPT, NDPT, and NPI.

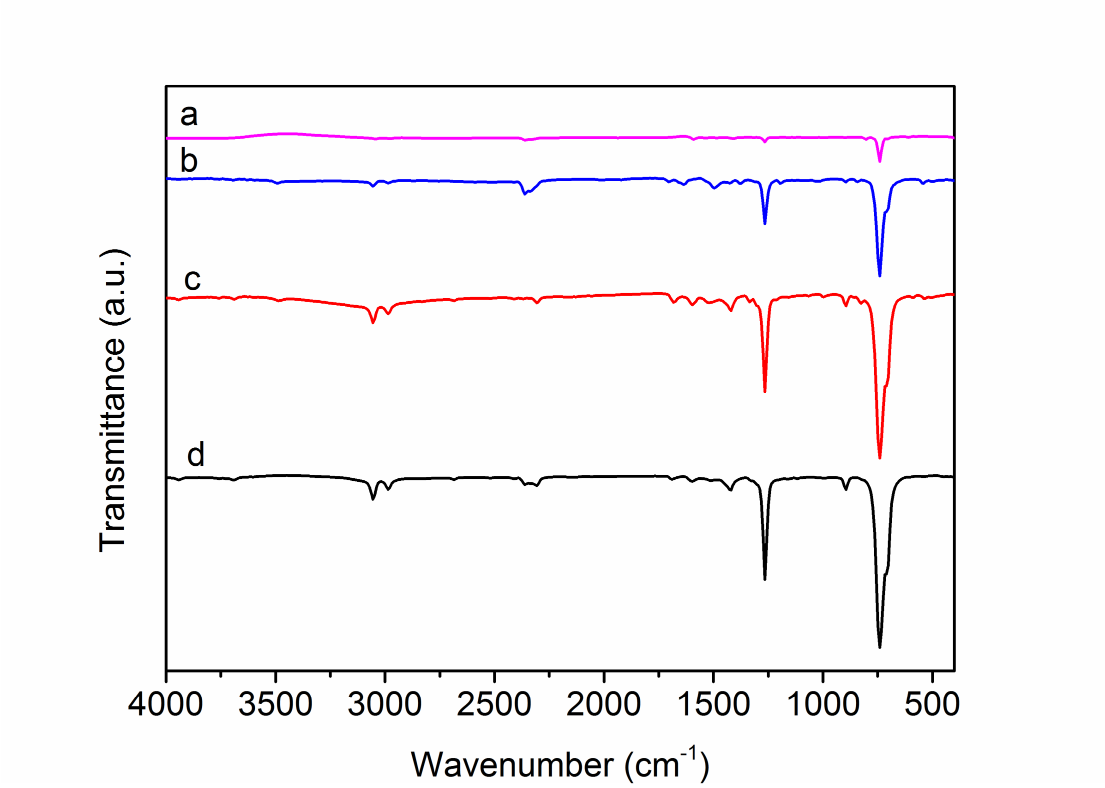

Figure S30. FT-IR spectra of (a) BPy, (b) DPT, (c) NPI, and (d) NDPT in DCM.

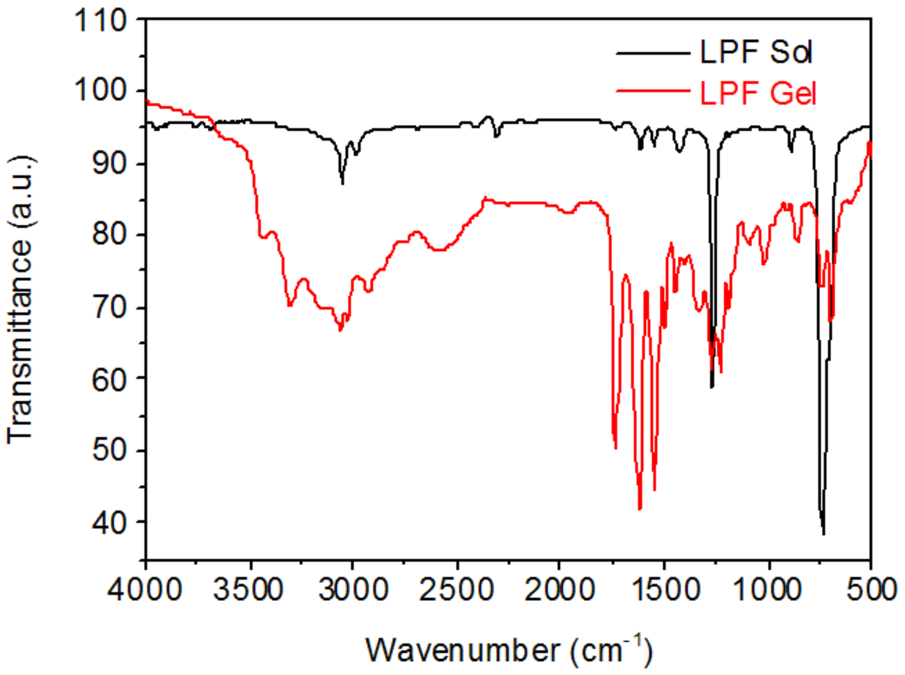

Figure S31. FT-IR spectra of LPF xerogel and LPF solution in DCM.

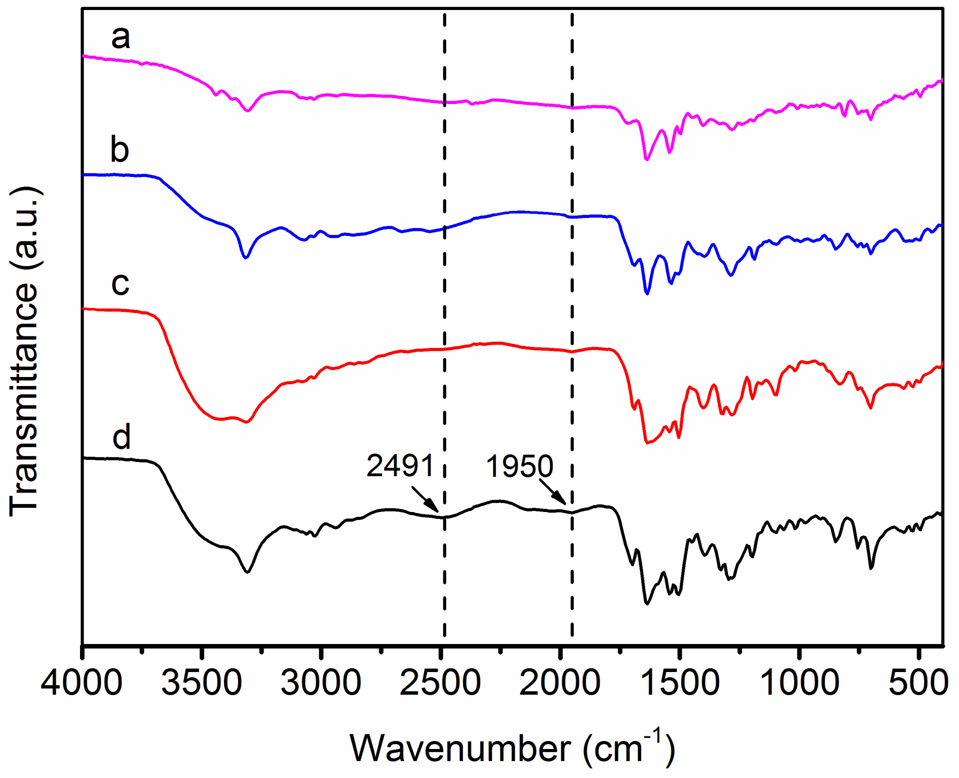

Figure S32. FT-IR spectra of (a) LPF+BPy, (b) LPF+DPT, (c) LPF+NDPT, and (d) LPF+NPI xerogels.

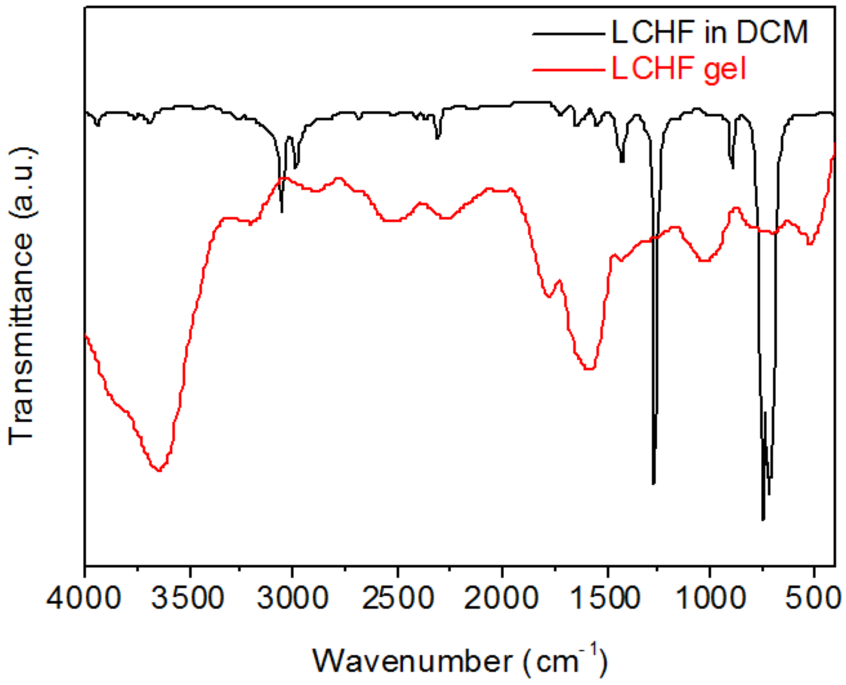

Figure S33. FT-IR spectra of LCHF xerogel and LCHF solution in DCM.

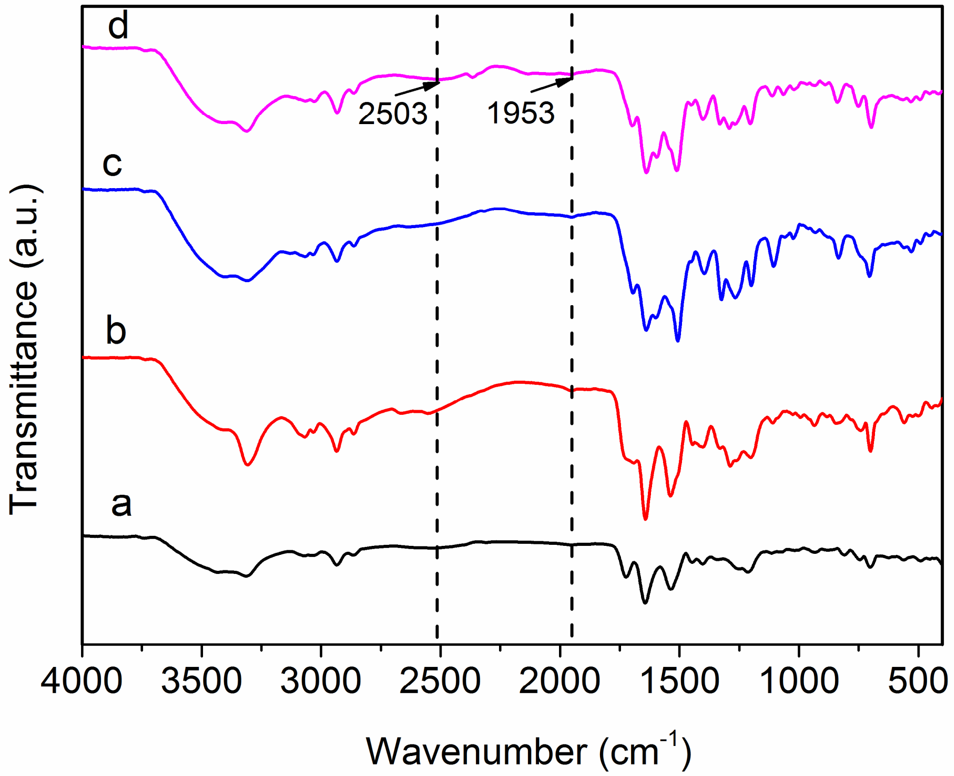

Figure S34. FT-IR spectra of (a) LCHF+BPy, (b) LCHF+DPT, (c) LCHF+NDPT, and (d) LCHF+NPI xerogels.

Table S1. Main vibrational bands (cm^{-1}) in FT-IR spectra of various samples (xerogels, powders, and solution). Spectra of solutions were measured by dropping DCM solutions on KBr wafers and were corrected for solvent and cell absorption. FT-IR spectra of solids were recorded after freeze-drying hydrogels over KBr pellets.

Assignment	ν_{NH}	ν_{OH} [a]	$\nu_{C=O}$	amide I	$\nu_{C=N}$ [b]	amide II	$\delta_{O\text{-}H}$	$\delta_{C=C}$ [c]
LPF Gel	3302		1738	1621		1551	1450	
LPF Sol			1740	1620		1550	1421	
LCHF Gel	3302		1726	1643		1537	1454	
LCHF Sol			1727	1638		1543	1424	
BPy Powder					1595			1040
								995
BPy Sol					1591			
LPF+BPy Gel	3308	2453	1713	1636		1543	1449	1010
		1951						
LCHF+BPy Gel	3313	2527	1722	1643		1535	1446	1018
								997
DPT Powder			1703					1013
								992
DPT Sol			1705					
LPF+DPT Gel	3317	2552	1690	1635		1536		1018
		1954						994
LCHF+DPT Gel	3310	2546	1732	1643		1537	1446	1024
		1951	1691					993
NDPT Powder			1690	1620	1596	1514		1020
								1000
NDPT Sol			1690		1601	1516		
LPF+NDPT Gel	3310	2495	1690	1635		1543		1019
		1950						
LCHF+NDPT Gel	3310	2566	1694	1639	1599	1541[d]	1450	1024
		1945						
NPI Powder			1688	1618	1592	1525		1004
								992
NPI Sol			1683		1596	1524		
LPF+NPI Gel	3310	2491	1697	1636	1588[d]	1543	1450	1019
		1950						
LCHF+ NPI Gel	3312	2503	1697	1638	1595	1543[d]	1450	1022
		1953						

[a] Stretching vibration of the hydroxy group in carboxylic acid-pyridyl hydrogen bonds. [b] Stretching vibration of the pyridine ring. [c] Twisting vibration of the pyridine ring. [d] Shoulder peak.

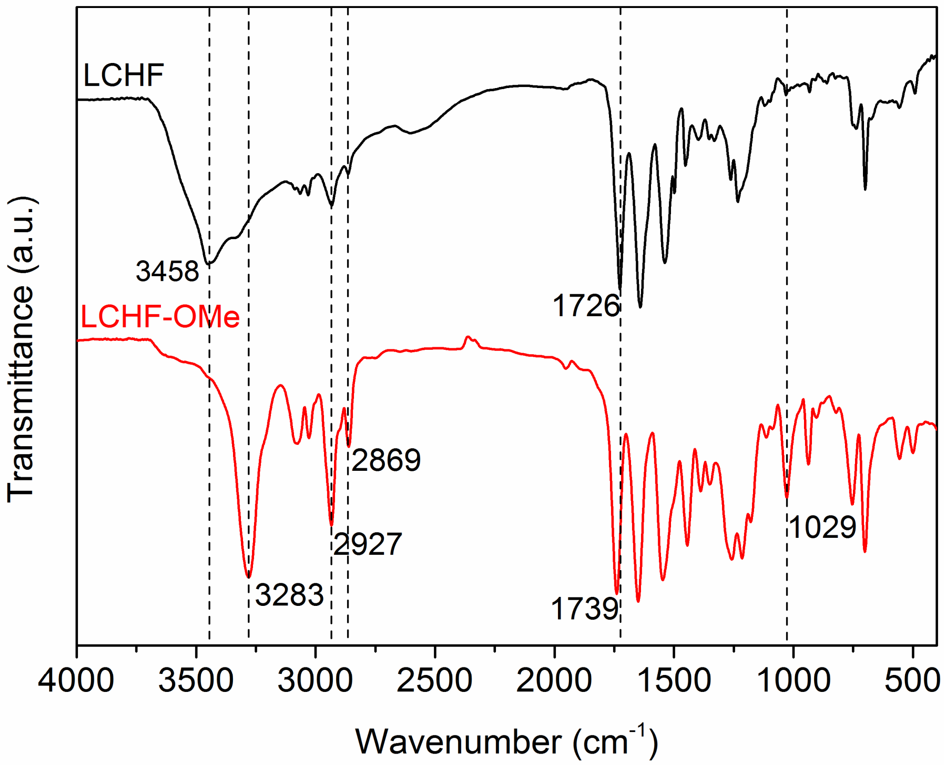

Figure S35. FT-IR spectra of LCHF powder and corresponding precursor LCHF-OMe. The FT-IR spectra of LCHF powder showed a strong and wide band around 3458 cm^{-1}, which is assigned to carboxylic group in LCHF. The FT-IR spectra of LCHF-OMe presented a well-defined band at 1029 cm^{-1}, which is attributed to the ester group. As Compared with the FT-IR spectrum of LCHF, the FT-IR spectrum of LCHF-OMe showed stronger bands at 2927 and 2869 cm^{-1} corresponding to the methyl group in LCHF-OMe. For LCHF-OMe powder, compared with LCHF exhibiting carboxylic band at 1726 cm^{-1} ($\nu_{C=O}$ of COOH), the carboxylic band of LCHF-OMe was observed at 1739 cm^{-1} ($\nu_{C=O}$ of $COOCH_3$).

Reference

S1. a) A. Tsuda, M. A. Alam, T. Harada, T. Yamaguchi, N. Ishii, T. Aida, *Angew. Chem. Int. Ed.*, **2007**, 46, 8198; b) M. Wolffs, S. J. George, Ž. Tomović, S. C. J. Meskers, A. P. H. J. Schenning, E. W. Meijer, *Angew. Chem. Int. Ed.*, **2007**, 46, 8203; c) P. Guo, L. Zhang, M. Liu, *Adv. Mater.*, **2006**, 18, 177; d) F. D. Saeva, G. R. Olin, *J. Am. Chem. Soc.*, **1977**, 99, 4848.
